# Convenient pH-responsive removal of Acid Black 1 by green l-histidine/iron oxide magnetic nanoadsorbent from water: performance and mechanistic studies†

**DOI:** 10.1039/c8ra09279f

**Published:** 2019-01-23

**Authors:** Qurrat-ul-Ain, Jaweria Khatoon, Muhammad Raza Shah, Muhammad Imran Malik, Iffat Abdul Tawab Khan, Sumaira Khurshid, Raheela Naz

**Affiliations:** Department of Chemistry, Faculty of Science, University of Karachi Karachi-75270 Pakistan qurrat_chem@uok.edu.pk +92 21 99261330 +92 21 99261300; Department of Chemistry, Federal Urdu University of Arts, Science and Technology, Gulshan-e-Iqbal Campus Karachi-75300 Pakistan; H. E. J. Research Institute of Chemistry, ICCBS, University of Karachi Karachi 75270 Pakistan raza.shah@iccs.edu

## Abstract

This study was aimed at developing green histidine-modified Fe_3_O_4_ nanoparticles (His-MNPs) for the adsorptive removal of Acid Black 1 (AB1) from aqueous solution. The His-MNPs were characterized by atomic force microscopy, scanning electron microscopy-energy dispersive X-ray spectrometry, infra-red spectra and thermogravimetry. These MNPs were spherical (average diameter 11–28 nm) with polydispersity index of 1.40 and about 13% mass coating of histidine. To optimize AB1 adsorption on His-MNPs and understand its mechanism, the influences of different operational variables (pH, adsorbent amount, temperature, initial AB1 concentration, contact time, ionic strength, *etc.*) on adsorption were examined with adsorption isotherms, kinetics and thermodynamic studies. The AB1 adsorption from water was fast with equilibrium time ≤ 45 min. The adsorption equilibrium was best fitted to the Langmuir isotherm model (*q*_max_ = 166.7 mg g^−1^ at the adsorbent dose of 0.2 g L^−1^, temperature 30 °C and pH 4). The linearity order for other isotherms was as follows: Dubinin–Radushkevich (D–R) < Temkin < Freundlich. The kinetics of the AB1 adsorption demonstrated the best compliance with the pseudo-second-order model, predominantly controlled by film diffusion as compared to intraparticle diffusion. Thermodynamic parameters (Δ*H*° and Δ*G*°) reflected the exothermic and spontaneous adsorption process. The values of Δ*G*°, Δ*H*°, activation energy and D–R free adsorption energy were all consistent with the physisorptive removal of AB1. The spectral (electronic and IR) and pH studies further corroborated the mechanism of AB1 removal by His-MNPs. The His-MNPs showed efficient adsorption, easy regeneration and excellent reusability, assisted by their pH-responsive properties. The prepared adsorbent can provide a safe, effective and economical alternative strategy for removing azo dyes from wastewater.

## Abstract 2

Effective and eco-friendly separation of Acid Black 1 dye from water by electrostatic adsorption onto reusable Histidine-Fe_3_O_4_ nanoparticles, credited to their pH-responsiveness.

## Graphical abstract

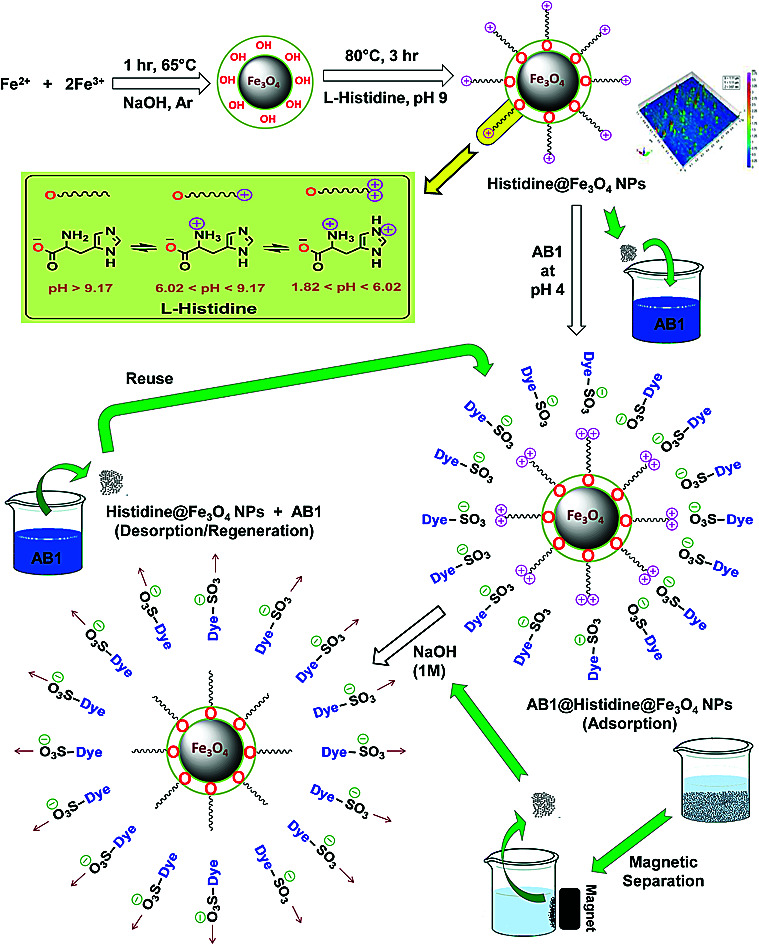

## Introduction

The availability of clean water is emerging as a major global challenge during this century, mainly due to a proportional increase in the number of industries and their wastewater.^1^ Among the various pollutants of wastewater, dyes are the most significant identified contaminants.^2^ They are heavily employed in diverse industries (textiles, cosmetics, leather, plastics, paper, construction, foodstuff, *etc.*) to color the related products.^3,4^ Inefficient and uneconomic dyeing practices may release up to 50% of dyes directly into waterways; thus, effluents from the dyeing industries are comprised of highly concentrated dyes as waste or wash liquor.^5^ The existence of these dyes in aquatic systems, even in very small amounts, is highly observable and undesirable for the environment and living species due to their high toxicity.^6^

Among all classes of dyes, azoic (–N=N–) dyes are the most difficult to biodegrade, possessing significant physicochemical, thermal and optical stability due to their stable aromatic ring structures.^7^ Certain azo dyes may release carcinogenic aromatic amines and mutagenic end products.^8^ They may cause skin allergy and affect the respiratory, reproductive and digestive systems of animals and humans.^9^ The color in water also reduces sunlight penetration and dissolved oxygen, posing a considerable threat to the aquatic biota.^6^ Therefore, the existence of toxic azo dyes is of environmental concern, and their removal is of great significance and an urgent need. Acid Black 1 (AB1), an anionic diazo dye, was selected for removal from water in the current study. AB1 is widely used in several cosmetic products including hair dye. AB1 is known for skin, eye and respiratory irritation, with acute or chronic toxicity.^10,11^

Among the various physical, chemical and biological treatment methods for removing dyes from wastewater, the adsorption technique is deemed as superior to others, because it is more simple and economical.^1,9^ A number of adsorbents have been studied for decolorizing wastewater, including polymers, activated carbon, zeolites, fly ash and nanoadsorbents.^6^ However, commercially available adsorbents suffer from the problems of secondary sludge waste increasing operation cost, long equilibrium time or low adsorption capacity.^12^ Therefore, significant efforts are needed for researchers to explore alternative, more efficient, adsorbents.

Recently, iron oxide nanoparticles, particularly magnetite nanoparticles (MNPs) with the formula FeO·Fe_2_O_3_ or Fe_3_O_4_, have emerged as new adsorbents in nanotechnology for wastewater treatment with many other applications, such as in biomedicine, materials science and biological sciences.^13^ The increasing interest in MNPs in environmental remediation is based on their merits of superparamagnetism, high surface area, large pollutant removal capacity, short diffusion route, fast reactivity, low cost, easy and fast magnetic separation, and surface modifiability. The surface modification of MNPs with a suitable material provides them protection from corrosion or agglomeration with additional physical and chemical functions (*e.g.*, biocompatibility, hydrophilicity, molecular conjugation and reduced toxicity) as compared to bare Fe_3_O_4_ nanoparticles.^14^ A wide range of organic and inorganic molecules, including polymers, surfactants and metallic species, have been used to provide suitable surface coating of MNPs for certain applications.^9,15,16^ However, the surface grafting of nanoparticles with biocompatible materials is only considered as a valuable approach in developing environmentally benign NPs with proven low toxicity.^14^

The coating of MNPs with amino acids is of increasing interest in recent years due to their very important biological role in the body, use in anticancer therapy, biocompatibility and good capping capability towards MNPs in the absence of toxic organic solvents.^17–19^ To date, many amino acids (*e.g.*, lysine, valine, arginine, glycine, glutamic acid, polyamino acid and serine) have been modified with iron oxide nanoparticles.^7,14,17,18,20–22^ However, only a few of them have been explored as nanoadsorbents to remove toxic azo dyes. Concerning this, l-lysine and l-glycine magnetite NPs have been employed to remove Orange 1 and Acid Red 18,^7,23^ and l-arginine-capped MNPs have been used to remove Reactive Blue 19.^4^ These reported amino acid-coated MNPs generally have low adsorption capacities (≤125 mg g^−1^) for azo dyes; therefore, other amino acids should also be studied to develop more efficient magnetic nanoadsorbents. In this regard, l-histidine (l-His) has been used in our present study to functionalize MNPs for the subsequent decontamination of harmful azo dyes. l-His is an essential α-amino acid of chemical formula C_6_H_9_N_3_O_2._ It is a nontoxic biomolecule involved in important biochemical, biomedical and metallic-enzymatic reactions.^24^ Besides having amino and carboxylic groups, it possesses an imidazole aromatic moiety in the side chain capable of varying its charge with pH, which could favor electrostatic interactions with anionic azo dyes. Due to the small size, l-His could easily stabilize Fe_3_O_4_ NPs with a positive effect on their growth and magnetic properties, providing efficient separation through magnetic decantation. l-His is an easily available and low-cost reagent. Keeping these properties in mind, l-His-Fe_3_O_4_ nanoparticles could be a very good choice as a sustainable adsorbent for efficiently removing hazardous anionic azo dyes from water/wastewater. To our knowledge, no previous study has, so far, been documented on AB1 removal by l-His-functionalized MNPs.

This work presents the synthesis of green l-His coated Fe_3_O_4_ NPs (His-MNPs) *via* coprecipitation, and the investigation of their potential to remove anionic azo dyes from water in batch adsorption experiments using AB1 as a model dye. The AB1 uptake capacity of His-MNPs adsorbent was determined as a function of different operational variables (such as dye concentration, temperature, pH, salt concentration and adsorbent dosage) to optimize the dye removal. The adsorption isotherms, kinetics and thermodynamics of His-MNPs for AB1 were studied to understand the adsorption mechanism and performance. Desorption of AB1 and the reusability of the synthesized adsorbent were also investigated.

## Experimental

### Materials

The reagents and chemicals utilized for the current work were of analytical grade and were used without any further purification. Acid Black 1 (dye content ∼ 85%) was procured from Sigma-Aldrich (Germany), and its important characteristics are provided in Table 1.

**Table:** Properties of Acid Black 1 (Naphthol blue black)

Dye symbol	Chemical structure	Chemical formula	Molecular mass (gmol^−1^)	*λ*_max_ (nm)
AB1	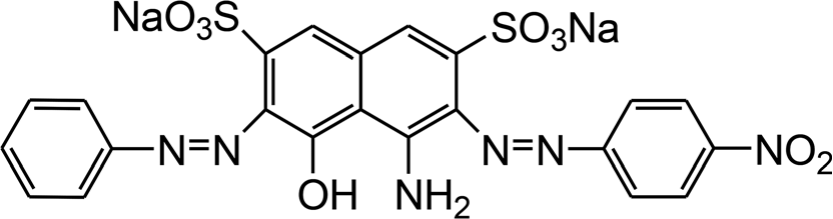	Na_2_C_22_H_14_N_6_O_9_S_2_	616.49	618

Iron metal salts (FeCl_3_·6H_2_O, FeSO_4_·7H_2_O), NaOH and l-histidine were purchased from Merck (Germany), and methanol from Tedia Company, Inc (USA). Acetic acid, NaCl and HCl were supplied by Honeywell (Germany). Distilled water was deionized using ELGA Cartridge Type C114 prior to preparing the required solutions.

### Synthesis of l-histidine-Fe_3_O_4_ nanoparticles

**Scheme 1:**
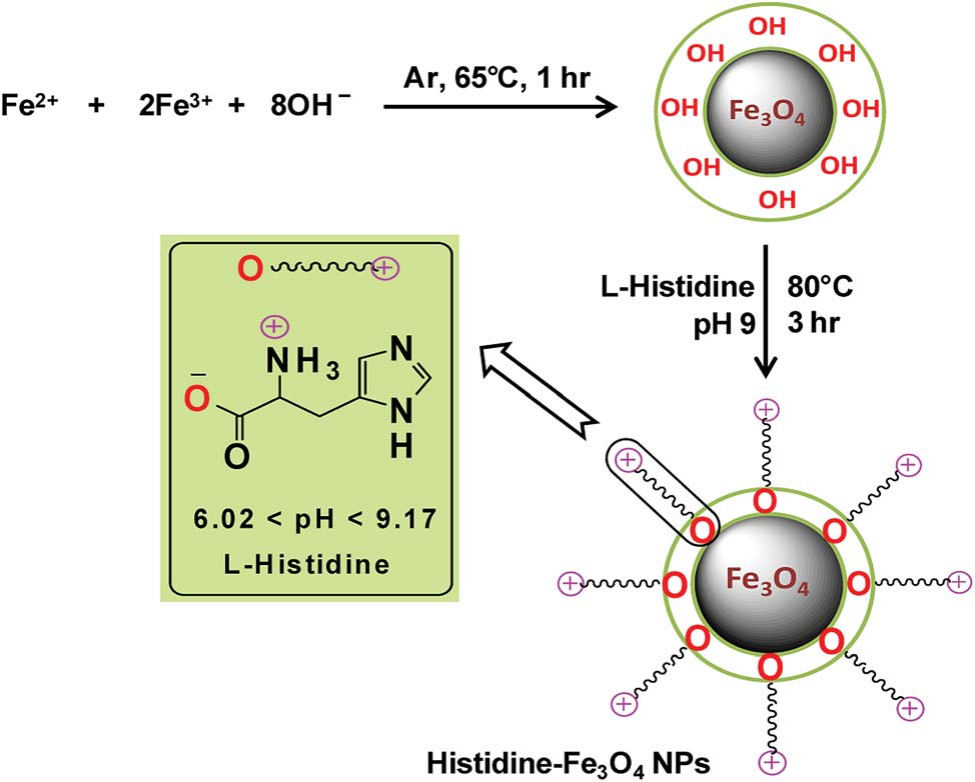
Schematic representation of the synthesis of His-MNPs.

The chemical co-precipitation method for the synthesis of bare Fe_3_O_4_ magnetic nanoparticles and their stabilization through surface modification with l-His in a post-synthesis step as described by Inbaraj and Chen (2012) were adopted, with some modifications, to synthesize l-His-Fe_3_O_4_ nanoparticles (His-MNPs).^20^ Briefly, an aqueous mixture of ferric chloride and ferrous sulphate (6.1 g and 4.2 g, respectively) was prepared in 100 mL of distilled-deionized water. The mixture was then heated to 65 °C with vigorous stirring under the bubbling of argon gas to prevent the unwanted oxidation of Fe^2+^ ions. Subsequently, 15 mL of 2.5 M NaOH solution was rapidly injected and the reaction mixture was continuously stirred at 65 °C under argon gas bubbling for an hour. The black precipitates of bare MNPs thus obtained were removed from solution through magnetic decantation and then washed many times with deoxygenated distilled-deionized water. For coating with l-His, bare MNPs were suspended in 40 mL of l-His solution (100 mM) prepared in hot deoxygenated distilled-deionized water. The solution pH was then fixed to 9 using a pH meter (HANNA, H12211). The dispersion was then sonicated in an ultrasonic bath (E30H ELMA, Germany) for 10 min. The mixture was refluxed further with vigorous stirring under argon gas at 80 °C for three hours to obtain well dispersed His-MNPs. The obtained product was separated using a powerful magnet, washed multiple times using hot distilled-deionized water until the supernatant became neutral and finally dried at 60 °C in a vacuum oven for 8 hours for further characterization. Scheme 1 presents the synthesis of His-MNPs with the possible mode of attachment of l-His and Fe_3_O_4_. Monodentate bonding of l-His to MNPs was confirmed by FT-IR studies. This bonding was sufficiently stable at pH 3 or higher. The carboxylate bonding at the His-MNPs surface also seemed viable at moderately higher temperatures, as revealed by sufficient adsorbing properties (79% dye removal) at about 90 °C under testing conditions.

### Characterization of His-MNPs

The vibrational spectra were collected using a Fourier transform infrared (FT-IR) spectrometer (Shimadzu IR-460) at 400–4000 cm^−1^ on KBr disks to determine the mode of bonding and surface functional groups. The dimensions and surface morphology of synthesized nanoparticles (bare and coated Fe_3_O_4_) were determined by atomic force microscope (AFM, model Agilent 5500) operated in tapping mode, and scanning electron microscope (SEM, model JSM-6380A, JEOL, Japan) using carbon-coated copper grids at an accelerating voltage of 20 kV. An energy dispersive X-ray spectrometry (EDS) detector (EX-54175jMU, Jeol, Japan) was also equipped with SEM to confirm the purity of the synthesized product. The sample was coated with 300 Å gold film for EDS analysis. The thermal stability of the synthesized nanoparticles and percent weight of l-His coated on Fe_3_O_4_ was determined by heating each sample (4–10 mg powder) in a thermogravimetric analyzer (SDT-Q600 V8.3 Build 101, USA) from 25–800 °C at a rate of 10 °C min^−1^ under nitrogen atmosphere. The surface charge (zeta potential) of His-MNPs was measured by a Zetasizer Nano ZS90 (Malvern Instruments, UK) using 0.5 g L^−1^ sample suspensions in deionized water at different pH values (2–13.5).

### Adsorption experiments

**Fig. 1:**
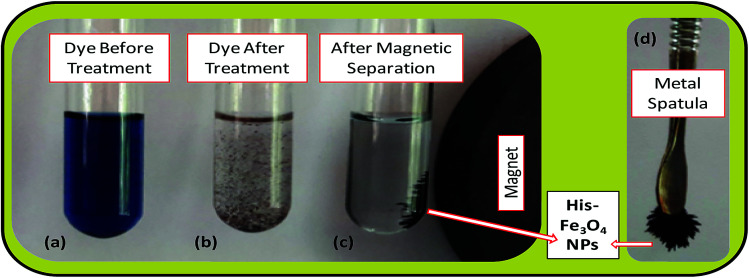
Real experimental photographs showing (a–c) the adsorptive removal of AB1 from water by His-MNPs, and (c and d) the magnetic nature of His-MNPs.

The batch-mode adsorption technique was used to remove AB1 from its aqueous solution by His-MNPs. Each adsorption experiment was performed in a 50 mL Erlenmeyer flask containing 30 mL of standard solution of AB1 (6.3 mg L^−1^) at the desired pH (4). To adjust the pH of the solution by pH meter, 0.1 M NaOH or 0.1 M HCl solution was used. The dye solution was contacted with a specified dried mass of His-MNPs, and the reaction mixture was shaken in a reciprocal shaking thermostatic water bath (SWB-A, BIOBASE) with a speed of 120 rpm at 30 °C for a certain time until adsorption equilibrium was established (50 min). At pre-determined time intervals, the AB1-loaded His-MNPs were quickly separated from unbound dye solution by magnetic decantation (using a powerful Nd-Fe-B magnet disk), and residual AB1 in solution was monitored by a UV-visible spectrophotometer (Shimadzu UV-240, Hitachi U-3200) at the maximum absorption wavelength of AB1 (*λ*_max_, 618 nm). Fig. 1 presents some real images showing the adsorptive elimination of AB1 from its aqueous solution through His-MNPs and magnetic separation of adsorbent. A calibration curve of absorbance (at *λ*_max_) *versus* AB1 concentration was plotted using standard model solutions of AB1 of pH 4 (linearity range 0–65 μM, *R*^2^ = 0.999) and applied to determine the unknown residual concentration of AB1 in solution.

For optimization of AB1 removal by His-MNPs, different operational parameters were studied in varying range to determine their effect on dye adsorption efficiency: pH (3–9), temperature (30–80 °C), salt concentration (0–0.8 M NaCl), adsorbent dosage (0.2–20 g L^−1^), initial AB1 concentration (0.7–40 mg L^−1^), and contact time (0–50 min). The effect on adsorption of soaking or impregnation of His-MNPs in the CH_3_OH–CH_3_COOH mixture (9 : 1 v/v) before use in the adsorption of AB1 was also investigated. Each experiment was carried out 3 times and average values (within ± 5% maximum deviation) were used for calculation. The AB1 removal efficiency (%) and adsorption capacity (*q*), *i.e.*, the amount of dye adsorbed onto His-MNPs (mg g^−1^) at different shaking times were computed using the following formulae:
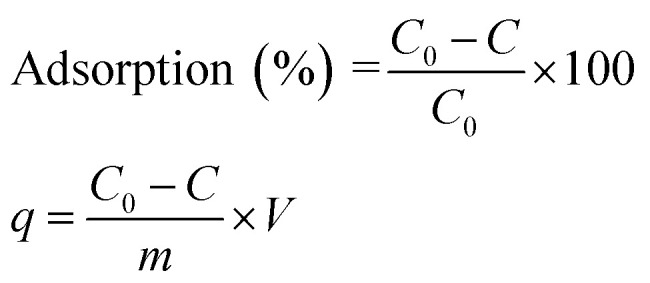
where *C*_0_ is the initial AB1 concentration (mg L^−1^), *C* is the residual AB1 concentration in the supernatant (mg L^−1^) at time *t* (min), *m* is the mass of His-MNPs (g) and *V* is the volume of the solution of AB1 (L). For equilibrium data, *C* replaces *C*_e_, and *q* replaces *q*_e_ in the above equations, where *C*_e_ and *q*_e_ are the concentration of AB1 in solution (mg L^−1^) and adsorption capacity of His-MNPs (mg g^−1^) at equilibrium, respectively. To demonstrate the AB1 adsorption process, common models of adsorption isotherms (Langmuir, Freundlich, Dubinin–Radushkevich, Temkin), kinetics (pseudo-first-order, pseudo-second-order, intraparticle diffusion, Boyd plot) and thermodynamics (Van't Hoff plot) were applied to the experimental data by the linear regression method using Microsoft Office Excel 2007 solver.

### Desorption and reusability experiments

For the recovery of dye from used His-MNPs, three different eluents (1 M NaOH, 1 M HCl, and 9 : 1 CH_3_OH : CH_3_COOH mixture) were used to find the best eluting solvent for desorption. An amount of used His-MNPs (obtained from 100 mg fresh His-MNPs) with a known amount of adsorbed AB1 were treated with a fixed volume (30 mL) of eluent at 30 °C and shaken for one hour to attain desorption equilibrium. After magnetic decantation, the AB1 concentration in the supernatant was determined and used to calculate the percentage of desorption. To evaluate the reusability potential of His-MNPs for AB1, the recycled His-MNPs (from NaOH) were washed with distilled water, dried and used again under conditions similar to the first adsorption/desorption cycle. This was repeated for up to 5 cycles.

## Results and discussion

### Characterization of His-MNPs

The bare and l-His coated Fe_3_O_4_ NPs, synthesized by the simple, economic and eco-friendly coprecipitation method,^13,20^ were characterized by FT-IR spectroscopy, AFM, SEM-EDS and TGA analyses. The synthesized NPs were magnetic in nature as shown by their quick response in a magnetic field (Fig. 1c and d).

#### FT-IR spectroscopy

**Fig. 2:**
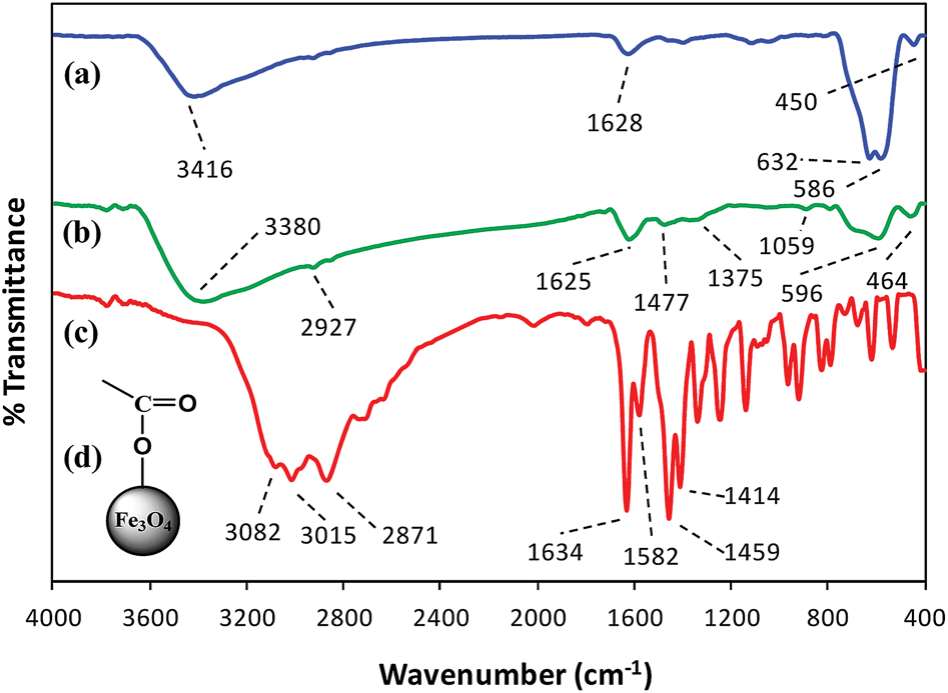
Comparative FT-IR spectra of (a) bare MNPs, (b) His-MNPs and (c) pure l-His. (d) The suggested monodentate carboxyl bonding mode of l-His on the MNPs surface.

FT-IR spectroscopy indicated the formation of l-His coated Fe_3_O_4_ NPs (His-MNPs) and its surface structure by comparing the FT-IR spectra of His-MNPs with bare MNPs and pure l-His (Fig. 2).

Two prominent bands at 3416 and 1628 cm^−1^ for bare MNPs (Fig. 2a) were assigned to the stretching and bending vibrations of O–H groups or water molecules coordinated to unsaturated Fe cations on the bare MNPs surface.^17^ The characteristic peaks of crystalline magnetite due to intrinsic Fe–O vibrations appeared at 586 cm^−1^ (split into two peaks, other at 632 cm^−1^) and 450 cm^−1^ for bare MNPs, and at 596 and 464 cm^−1^ for His-MNPs (Fig. 2b).^18^ A significant decrease in the peak intensity at 586 cm^−1^ after coating reflects the adsorption of l-His on the MNPs surface.^15^ The small peak at 2927 cm^−1^ corresponds to the stretching vibration of C–H in His-MNPs. A broad band originating at 3380 cm^−1^ in the FT-IR spectrum of His-MNPs is a result of the overlapping of O–H and amino N–H stretching vibrations.^4,7^ In the pure l-His spectrum (Fig. 2c), the distinct peaks attributed to amine N–H stretching, asymmetric deformation (*δ*_as_) and symmetric deformation (*δ*_s_) exist at 3082, 1582 and 1459 cm^−1^, respectively, and aliphatic C–H stretching was observed at 3016 cm^−1^. The peak at 2871 cm^−1^ is ascribed to overtones and combinational tones.^25^ The pure l-His also exhibits two strong peaks at 1414 and 1634 cm^−1^ because of symmetric and asymmetric stretching vibrations (*ν*_s_ and *ν*_as_), respectively, of the carboxyl (COO^–^) group.^26^ The *ν*_as_ (COO^–^) and *ν*_s_ (COO^–^) stretching vibrations of coordinated l-His in His-MNPs were observed at 1625 cm^−1^ (overlapped with amine *δ*_as_) and 1375 cm^−1^, respectively.^20,25^ The reduction in the intensity of the carboxyl group peaks of His-MNPs as compared to pure l-His is due to carboxyl interaction with surface OH groups,^18^ and the broadening or shifting of carboxyl peaks refers to l-His adsorption on the NPs surface *via* carboxyl groups.^27^ Moreover, the difference between asymmetric and symmetric stretching frequencies of carboxyl groups for His-MNPs (Δ*ν*_as–s_ = 250 cm^−1^) is higher than that for uncoordinated l-His (Δ*ν*_as–s_ = 220 cm^−1^), which demonstrates the monodentate bonding mode of the COO^–^ group with the iron cation as shown in Fig. 2d.^19^ Additionally, the overlapped, broadened and significantly reduced intensity band of imidazole ring vibrations occurring at 1059 cm^−1^ in the His-MNPs spectrum suggests the involvement of the imidazole moiety in the binding of l-His to His-MNPs. The peak corresponding to amine *δ*_s_ in His-MNPs is also visible at 1477 cm^−1^. The presence of typical peaks corresponding to magnetite and l-His in the FT-IR spectrum of His-MNPs reasonably indicate the successful modification of MNPs with l-His.

#### Size and morphology

**Fig. 3:**
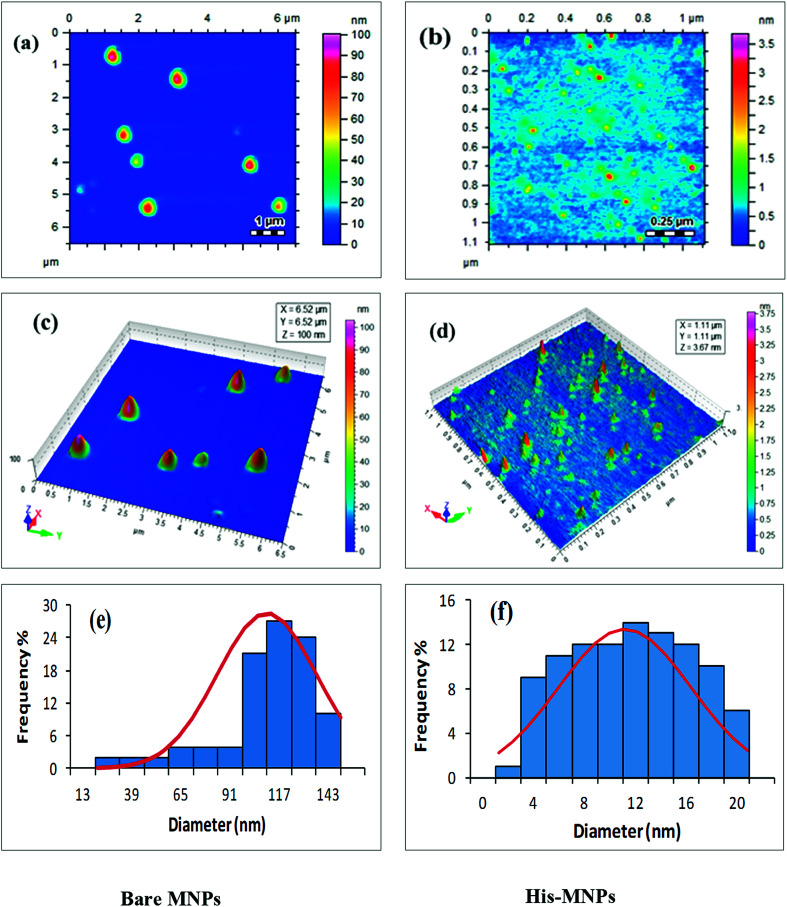
AFM analysis of bare MNPs (left) and His-MNPs (right). (a and b) 2D topographic images, (c and d) 3D topographic images, and (e and f) particle size distribution.

The physical and chemical properties of NPs are greatly affected by their size and shape. The effect of l-His coating on the morphology and particle size of MNPs was investigated by atomic force microscopy (AFM). Highly resolved two-dimensional and three-dimensional AFM topographic images of bare MNPs and His-MNPs were obtained as shown in Fig. 3. AFM analysis revealed the existence of polydisperse magnetic nanoparticles of varying sizes. The value of the polydispersity index (PDI) of nanoparticles was calculated by the following equations as described by Nematollahzadeh *et al.* (2012):^28^PDI = *D*_w_/*D*_n_*D*_w_ = Σ(*d*_i_)^4^/Σ(*d*_i_)^3^*D*_n_ = Σ*d*_i_/*n*where *D*_n_, *D*_w_, *d*_i_ and *n* represent the number-average diameter, weight-average diameter, diameter and number of nanoparticles, respectively. The size count for a total of 100 randomly selected particles of each sample from AFM micrographs was used to measure the polydispersity. The PDI value was 1.13 for bare MNPs and 1.40 for His-MNPs, showing no major difference in the homogeneity of the particle size distribution between bare and coated MNPs. The 3D images clearly showed isolated NPs with aggregates having a nearly spherical shape. The bare MNPs demonstrated grain diameter sizes in the range of 14 to 143 nm with an average diameter of 104 nm (Fig. 3e). In contrast, the l-His coated MNPs showed significantly reduced particle sizes and narrower size distribution in the range of 2–20 nm with an average diameter of 11 nm (Fig. 3f). The decreased particle size of MNPs after surface modification with l-His is likely due to reduced agglomeration.

**Fig. 4:**
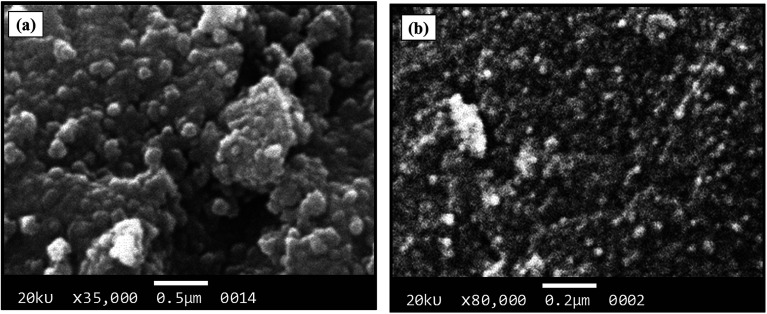
SEM images of (a) bare MNPs and (b) His-MNPs.

**Fig. 5:**
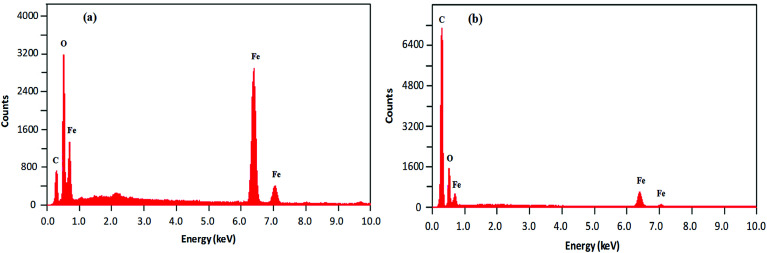
SEM-EDS analysis results of (a) bare MNPs and (b) His-MNPs.

The morphological analysis of magnetic NPs was also carried out by scanning electron microscopy (SEM). The SEM photomicrographs and SEM–energy dispersive X-ray spectra (SEM–EDS) of MNPs (before and after surface modification) are depicted in Fig. 4 and 5, respectively. The SEM images showed spherical nanocrystal clusters or aggregations due to magnetic forces between the nanoparticles.^29^ The SEM diameters of isolated bare MNPs and His-MNPs were found to be 59–140 nm (average diameter = 107 nm) and 20–40 nm (average diameter = 28 nm), respectively. The larger clusters of bare MNPs as compared to His-MNPs indicate that surface coating with l-His could stabilize MNPs, which might suppress their aggregation.^30^ Thus, the results of AFM and SEM are in close agreement with each other. The SEM–EDS spectra (Fig. 5) provided the chemical composition and elemental percentages of magnetic NPs as follows: Fe, 59.1%; O, 22.6%; C, 18.3% for bare MNPs, and Fe, 9.30%; O, 32.1%; C, 58.6% for His-MNPs. The low-intensity carbon peak observed for bare MNPs was due to carbon-coated grids used in EDS analysis,^10^ while the remaining elemental percentages fairly indicate the Fe_3_O_4_ composition. A significant decrease in the iron peak with a considerable enhancement of the carbon peaks in the EDS spectrum of His-MNPs compared to that of bare MNPs confirms the existence of the l-His functionality on the surface of MNPs. The Fe/O counts ratio from EDS for bare MNPs (0.75) is about 9 times higher as compared to the Fe/O counts ratio of His-MNPs (0.083), which also confirms the l-His attachment to MNPs. The absence of any other signal in the EDS detection limits confirms the purity of the synthesized magnetite products.

#### Thermal analysis

**Fig. 6:**
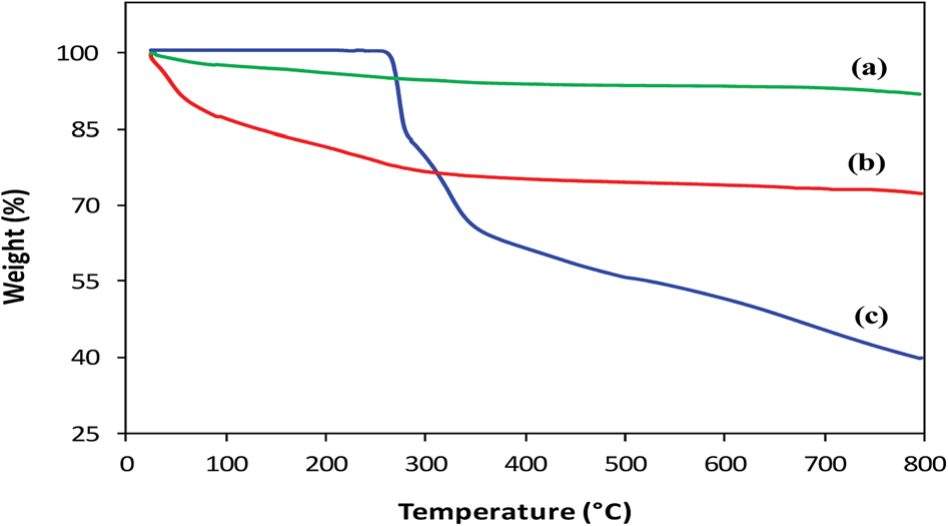
TGA thermograms of (a) bare MNPs, (b) His-MNPs and (c) pure l-His.

Thermal Gravimetric Analysis (TGA) was performed to confirm coating, estimate the amount of l-His loaded on MNPs and compare the thermal degradation behavior of His-MNPs with precursors. The TGA thermograms of bare MNPs, His-MNPs and pure l-His are shown in Fig. 6. The bare MNPs (Fig. 6a) possess high thermal stability, showing a small weight loss of 8.2% in the TG temperature range (25–800 °C) with significant mass reduction (about 66% of total weight loss) before 300 °C. The initial weight loss of about 2–3% around 100 °C and then the increase to about 5% near 300 °C for bare MNPs are attributed to the liberation of physisorbed and chemisorbed water, respectively.^31^ This corresponds to the molar ratio of about 4 : 3 between Fe_3_O_4_ and adsorbed water. The subsequent slight reduction in percent weight from 95 to 91.8% between 300 to 800 °C for bare MNPs is possible due to the decomposition of magnetite to non-stoichiometric magnetite according to the following reaction: Fe_3_O_4_ → Fe_3_O_4−*x*_ + (*x*/2)O_2_. A higher mass loss observed for His-MPs (Fig. 6b) as compared with bare MNPs, *e.g.*, four times higher percent weight loss (27.8%) at 800 °C, provides evidence of the l-His layer/coat at the His-MNPs surface. The pure l-His (Fig. 6c) is thermally stable up to 260 °C; however, further heating results in considerable weight loss (60.5% at 800 °C) through improperly resolved multi-stage decomposition processes. The first sharp mass loss for pure l-His (260–290 °C) occurs around its decomposition point (281 °C). This step causes 18.4% weight loss, which is effectively consistent with the release of CO gas from the carboxyl group of l-His. Two other apparent mass losses at about 300 and 500 °C can be assigned to the decomposition of the imidazole ring.^32^ From Fig. 6, it appears that the degradation of coated l-His over His-MNPs occurs at a much lower temperature as compared to pure l-His, probably because of the strong catalytic effect of Fe_3_O_4_ NPs on the thermal degradation of l-His, making its degradation faster. Such a catalytic effect was also observed by Durmus *et al.* (2009) for l-lysine coated MNPs.^19^ The 11.9% initial weight loss observed for His-MNPs before 100 °C is ascribed to the discharge of water molecules adsorbed on the surface. The subsequent sharp mass loss of 11.3% up to 300 °C followed by another mass loss of 2.1% up to 500 °C due to carboxyl and imidazole decomposition, respectively, indicate the involvement of carboxyl and imidazole groups in the binding of l-His to the MNPs surface.^31,32^ This is in agreement with the coordination behavior established by FT-IR spectra. Unlike bare and coated MNPs that show almost a mass plateau after 600 °C, thermal degradation of pure l-His does not stop over the TG temperature range, as revealed by about 3% weight loss within the last 50 °C of the TG window. Therefore, the 39.5% weight residue at 800 °C yielded by pure l-His may be comprised of incompletely degraded l-His and coke produced under a pyrolytic nitrogen environment.^19^ Based on the relative comparison of TGA weight losses (WL) of bare and l-His coated MNPs at 800 °C, the percent coated mass of l-His on MNPs was estimated as 13% (assuming zero coke weight) or higher (if some coke weight) using the following calculations:% *W*_(coated l-His)_ = % WL_(His-MNPs)_ − [% WL_(H_2_O, His-MNPs)_ + (% WL_(Fe_3_O_4_)_ − WL_(H_2_O, Fe_3_O_4_)_)] = 27.8 − [11.9 + (8.2 − 5)] = 13%

### Dye adsorption analysis

To optimize the batch mode adsorption process of AB1 by His-MNPs, the effect of varying different parameters that can affect the AB1 adsorption in aqueous solution was investigated.

#### Effect of pH

**Fig. 7:**
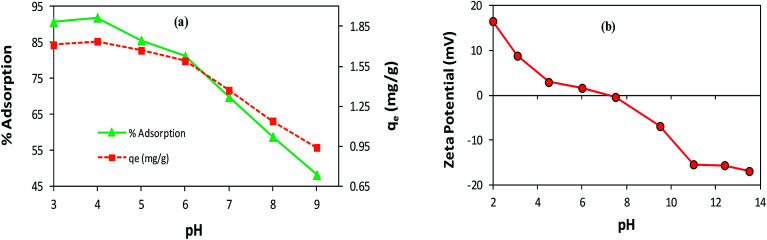
(a) Effect of pH on the adsorption of AB1 by His-MNPs. (b) Zeta potential analysis of His-MNPs.

The variation in pH alters the surface charge and dissociation of the functional groups of the adsorbent/adsorbate, which may drastically affect the adsorption process.^9^ To investigate the pH effect on AB1 adsorption by His-MNPs, the pH of the solution of AB1 was varied over a range of 3–9 (by diluted HCl and NaOH solutions), while other variables were kept constant (temperature, 30 °C; adsorbent amount, 3.3 g L^−1^; dye concentration, 6.3 mg L^−1^; equilibrium stirring time, 50 min). As shown in Fig. 7a, the value of the equilibrium percent adsorptive removal and adsorption capacity (*q*_e_) was highest at pH 4 (91.92% and 1.738 mg g^−1^, respectively), noticeably decreasing with increasing pH up to 9, reaching 48.20% and 0.941 mg g^−1^, respectively. This indicates that pH is a critical limiting factor for the AB1 removal process, and therefore pH 4 was selected as the optimized pH for further study. The adsorptive removal at pH 3 (90.70%, 1.711 mg g^−1^) was slightly lower as compared with that observed at pH 4, probably due to the detachment of some l-His from the magnetite surface resulting from the protonation of a carboxyl group,^54^ or the decomposition of MNPs at lower pH values.^33^ The significantly better adsorption efficiency of His-MNPs at acidic pH as compared to basic pH suggests that the adsorption is due to the electrostatic attraction between His-MNPs (cationic) and AB1 dye (anionic). These ionic interactions have been discussed in detail under the mechanism section of this article. The observed effect of pH on AB1 adsorption is consistent with zeta potential measurements of His-MNPs at various pH values (Fig. 7b). The pH of the zero point charge (pH_zpc_) for His-MNPs was within 7–7.5. At pH < pH_zpc_ the surface of His-MNPs had a positive charge (positive ζ-potential) due to the protonated amines of l-His, favoring the adsorption of anionic AB1 in an acidic environment. Conversely, at pH > pH_zpc_, the deprotonation of the amine and carboxyl groups of l-His gives a negative charge (negative ζ-potential) to the His-MNPs surface, rendering increased repulsion and decreased adsorption of AB1 at the adsorbent surface in basic medium.

#### Effect of soaking His-MNPs in an organic solvent mixture

A certain dried mass of His-MNPs (corresponding to adsorbent dosage of 3.3 g L^−1^ and 0.2 g L^−1^) was soaked in a mixture of acetic acid and methanol (1 : 9) for 25 min, air dried and then used as His-MNPs(s) for the adsorption of AB1 to study the effect of prior treatment with organic solvents, if any, on the adsorption capacity of magnetic nanoparticles. The initial AB1 concentration during this study was 6.3 mg L^−1^ (for adsorbent dose of 3.3 g L^−1^) and 24.7 mg L^−1^ (for adsorbent dose of 0.2 g L^−1^). The other experimental parameters were kept constant (pH 4, temperature 30 °C and equilibrium stirring time of 50 min). Table 2 shows a significant difference in the equilibrium percent removal and adsorption capacities (*q*_e_) of His-MNPs(s) and His-MNPs under the stated conditions.

**Table:** Comparison of the adsorption efficiency of His-MNPs and His-MNPs(s) against AB1

S. no.	Adsorbate concentration (mg L^−1^)	Adsorbent dosage (g L^−1^)	His-MNPs		His-MNPs(s)^a^	
			% adsorption	*q*_e_ (mg g^−1^)	% adsorption	*q*_e_ (mg g^−1^)
1	6.3	3.3	91.92	1.738	99.80	1.940
2	24.7	0.2	80.90	92.82	92.37	106.0

a His-MNPs adsorbent treated with acetic acid-methanol (1 : 9) mixture before use.

It is clearly evident from Table 2 that the His-MNPs were activated and their efficiency against AB1 increased after treatment with the organic solvent mixture. It is possible that the organic solvent molecules may be incorporated into the internal framework of the magnetic nanoparticles with some swelling effects and break some inter-particle magnetic attraction, enabling the further penetration of large dye molecules and providing more open surfaces or exposed active sites available for adsorption. Considering this effect, both His-MNPs and His-MNPs(s) were utilized in subsequent studies for evaluating the effect of the initial adsorbate concentration on adsorption for further comparison and optimization of AB1 removal.

#### Effects of other variables (temperature, electrolyte, adsorbent dosage, AB1 concentration, contact time)

The experimental data and detailed description of the study of the effects of temperature, electrolyte concentration, His-MNPs dosage, initial AB1 concentration and contact time on AB1 adsorption are provided in the ESI.† AB1 removal was optimum at 30 °C and decreased with increasing temperature, suggesting exothermic adsorption (Fig. S1†). The presence of NaCl did not influence AB1 adsorption at pH 4 and 6.2, even at higher salt concentrations (Fig. S2†). The amount of His-MNPs was positively related to the AB1 removal efficiency (%), and negatively to *q*_e_; the adsorption efficiency reached 99.2% at 20 g L^−1^ adsorbent, pH 4, 30 °C and 6.3 g L^−1^ dye (Fig. S3†). AB1 adsorption was also a function of AB1 concentration; the trend of percent removal and *q*_e_ on varying concentration was opposite to that observed on varying the His-MNPs dosage (Fig. S4†). This study yielded a maximum experimental *q*_e_ of 152.5 mg g^−1^, which was higher as compared to many previously reported magnetite sorbents.^9,12,38^ Contact time study revealed the attainment of adsorption equilibrium within 45 min or less (Fig. S5 and S6†). Fast AB1 removal by His-MNPs within a short contact time indicates that film diffusion is a predominant step as compared to intra-particle diffusion.^9^ Alteration of each variable in the tested range somehow affected the equilibrium time of AB1 adsorption by His-MNPs (Fig. S6†). Increasing pH from 3 to 9, adsorbent dose from 0.2 to 20.0 g L^−1^ and NaCl concentration from 0.0 to 0.8 M gradually increased the equilibrium time of adsorption from 15 to 45 min. In contrast, varying the temperature from 30 to 80 °C and dye concentration from 0.7 to 40 mg L^−1^ rendered somewhat faster equilibrium, going from 27 to 21 min and 35 to 15 min, respectively. The pH can change the surface charge and degree of ionization, thus modifying the ionic interactions and liquid film diffusion rates during AB1 adsorption. Additionally, the variation in the solution temperature, AB1 concentration and His-MNPs dosage may affect the steric or driving forces involved in AB1 diffusion processes on, or inside, magnetic His-MNPs, which may be present with some agglomeration (ζ-potential within ±20 mV). Hence, the rates of adsorption of AB1 could not be ascribed as being controlled by any single phenomenon.

### Adsorption equilibrium

**Fig. 8:**
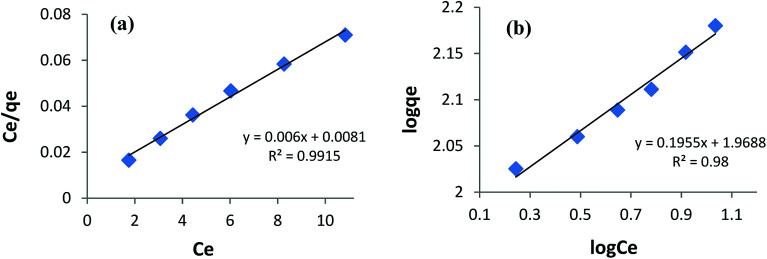
(a) Langmuir adsorption isotherm and (b) Freundlich adsorption isotherm for the removal of AB1 with His-MNPs.

**Fig. 9:**
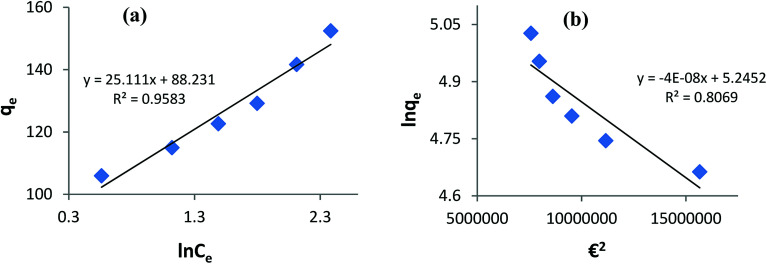
(a) Temkin adsorption isotherm and (b) Dubinin–Radushkevich adsorption isotherm for the removal of AB1 with His-MNPs.

Fitting the equilibrium adsorption data to the most appropriate isotherm model has fundamental importance in designing an optimized adsorption system for dye removal. In the current study, the experimental equilibrium data for the adsorption of AB1 onto His-MNPs were analyzed by using four isotherm models: Langmuir, Freundlich, Temkin and Dubinin–Radushkevich (Fig. 8 and 9). The expressions of the linear forms of all four adsorption isotherms are given in Table 3. These isotherms establish the relationship between the equilibrium concentration of the adsorbate in the bulk liquid phase (*C*_e_, mg L^−1^) and the dye uptake per unit mass of adsorbent (*q*_e_, mg g^−1^) at a constant temperature. The respective adsorption parameters and correlation coefficients (*R*^2^) calculated from all the isotherm plots for AB1 adsorption onto His-MNPs are summarized in Table 4. These adsorption isotherm studies were conducted under optimum conditions of pH, temperature and adsorbent dosage (4, 30 °C and 0.2 g L^−1^, respectively) with six different initial dye concentrations (24.7, 27.8, 30.8, 33.9, 37 and 40 mg L^−1^).

**Table:** Applied adsorption isotherms and their linear forms

Isotherm model	Linear form	Plot	Reference
Langmuir	*C*_e_/*q*_e_ = 1/(*q*_max_*K*_L_) + (1/*q*_max_)*C*_e_	*C*_e_/*q*_e_*vs. C*_e_	4
Freundlich	log *q*_e_ = log *K*_F_ + (1/*n*)log *C*_e_	log *q*_e_*vs.* log *C*_e_	40
Temkin	*q*_e_ = (*RT*/*b*)ln *A* + (*RT*/*b*)ln *C*_e_	*q*_e_ *vs.* ln *C*_e_	9
Dubinin–Radushkevich	ln *q*_e_ = ln *q*_m_ − *K*_DR_*€*^2^	ln *q*_e_*vs. €*^2^	41

Calculated parameters of isotherms for AB1 adsorption onto His-MNPs^a^

**Table:** 

Langmuir isotherm				Freundlich isotherm		
*q*_max_ (mg g^−1^)	*K*_L_ (L mg^−1^)	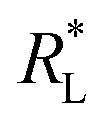	*R* ^2^	*K*_F_ (mg^1−1/*n*^ L^1/*n*^ g^−1^)	*n*	*R* ^2^
166.7	0.741	0.035	0.992	93.07	5.115	0.980

a Experimental conditions: pH = 4, adsorbent dosage = 0.2 g L^−1^, temp. = 30 °C.

**Table:** 

Dubinin–Radushkevich isotherm				Temkin isotherm		
*q*_m_ (mg g^−1^)	*K*_DR_ (mol^2^ J^−2^)	*E* (kJ mol^−1^)	*R* ^2^	*A* (L g^−1^)	*b* (J mol^−1^)	*R* ^2^
189.6	4 × 10^−8^	3.535	0.807	33.57	100.3	0.958

#### Langmuir isotherm

The Langmuir isotherm model is applicable to the saturated monolayer coverage of adsorbate species on the homogeneous surface of the adsorbent with constant energy and no lateral interaction between adsorbed species.^4,6^ The constants *q*_max_ and *K*_L_ in the linear expression of the Langmuir isotherm (Table 3) describe the maximum adsorption capacity (mg g^−1^) of the adsorbent for monolayer, and energy/heat of adsorption (L mg^−1^), respectively. The Langmuir adsorption equilibrium constant (*K*_L_) is associated with binding site affinity. The values of *K*_L_ (0.741 L mg^−1^) and *q*_max_ (166.7 mg g^−1^) were determined from the intercept and slope of the linear plot of *C*_e_/*q*_e_*versus C*_e_ (Fig. 8a). The calculated *q*_max_ is in close agreement with the real experimental value (*q*_exp_ = 152.5 mg g^−1^). An essential characteristic of the Langmuir isotherm is a dimensionless constant (*R*_L_) called the separation factor (*R*_L_ = 1/(1 + *K*_L_*C*_0_), wherein *C*_0_ is the initial dye concentration in mg L^−1^). The value of *R*_L_ varied from 0.052 to 0.033 with initial concentration change in AB1 dye from 25 to 40 mg L^−1^ at the studied temperature. This suggests a favorable adsorption process between AB1 and His-MNPs as 0 < *R*_L_ < 1.^39^ The Langmuir isotherm yields the highest correlation coefficient (*R*^2^ = 0.992, almost equal to unity) among all models (Table 4), suggesting that the best fit of the AB1 adsorption equilibrium is to the Langmuir model.

#### Freundlich isotherm

The Freundlich isotherm model presumes multilayer adsorbate adsorption on a heterogeneous surface of an adsorbent having diverse energy sites and affinities with mutual interaction of adsorbed molecules.^33,40^ The linear Freundlich isotherm of log *q*_e_*versus* log *C*_e_ (Fig. 8b and Table 3) gives the Freundlich constant (*K*_F_) and heterogeneity factor (*n*). The constants *n* (unitless) and *K*_F_ (mg^1−1/*n*^ L^1/*n*^ g^−1^) are the measure of intensity/strength of adsorption and adsorption capacity, respectively. The values of *n* and *K*_F_ calculated from the Freundlich isotherm were 5.115 and 93.07, respectively (Table 4). The value of *n* within 2–10 supports favorable adsorption and effective interaction between AB1 and His-MNPs.^36^ The corresponding regression coefficient value (*R*^2^ = 0.980) reveals a good fit of the experimental equilibrium data to the Freundlich isotherm model but a poorer fit as compared to the Langmuir model (*R*^2^ = 0.992).

#### Temkin isotherm

The Temkin isotherm model assumes a linear fall in the heat of adsorption of molecules in the layer as a function of temperature and a uniform distribution of binding energies.^9^ It investigates the heat of adsorption and binding interactions. The heat of adsorption is related to the Temkin constant *b* (J mol^−1^), which is calculated from the slope of the linear plot of *q*_e_*versus* ln *C*_e_ (Fig. 9a). The intercept of this Temkin isotherm plot gives an equilibrium binding constant (*A*, L g^−1^) that is related to the maximum binding energy. *T* and *R* in the linear Temkin equation (Table 3) are absolute temperature (Kelvin) and universal gas constant (8.314 J mol^−1^ K^−1^). The estimated values of *b*, *A* and *R*^2^ for the Temkin model (Table 4) were found to be 100.3 J mol^−1^, 33.57 L g^−1^ and 0.958, respectively. The regression coefficient shows that the Temkin model does not appropriately fit to experimental adsorption data as compared to the Freundlich and Langmuir models.

#### Dubinin–Radushkevich isotherm

The Dubinin–Radushkevich (D–R) isotherm model describes the variation in adsorption potential during the adsorption process and surface heterogeneity.^9,41^ The slope and intercept of the linear D–R isotherm between ln *q*_e_ and *€*^2^ (Table 3 and Fig. 9b) determine the adsorption energy-related constant *K*_DR_ (mol^2^ J^−2^) and theoretical adsorption capacity *q*_m_ (mg g^−1^). The Polanyi potential (*€*) is equal to *RT*(1 + 1/*C*_e_). The constants *K*_DR_ and *q*_m_ were calculated as 4 × 10^−8^ mol^2^ J^−2^ and 189.6 mg g^−1^, respectively (Table 4). The constant *K*_DR_ is used to determine the mean free adsorption energy (*E*) required for one mole of dye to transfer from the solution to the solid surface using the following equation:
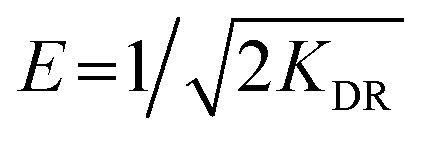


The value of *E* predicts the type of adsorption process as physical (*E* < 8 kJ mol^−1^), chemisorption (*E* > 16 kJ mol^−1^) or chemical ion exchange (*E* = 8–16 kJ mol^−1^).^42^ The mean adsorption energy of 3.535 kJ mol^−1^ (Table 4) suggests that the AB1 adsorption onto His-MNPs is mainly accompanied by physisorption. The regression coefficient for the D–R model is 0.807, suggesting that the experimental adsorption data are not fitted to this model. Hence, the linearity of four isotherm models for AB1 adsorption onto His-MNPs was in the order of Dubinin–Radushkevich (D–R) < Temkin < Freundlich < Langmuir (Table 4). It portrays the monolayer adsorption of AB1 dye with a homogeneously distributed finite number of identical active sites on the His-MNPs surface. The reported literature reveals a great scarcity of research data regarding the adsorption of AB1 onto Fe_3_O_4_-based adsorbents. However, the studies of AB1 adsorption with other traditional adsorbents have been found significant; some of them are reported in Table 5 with adsorption capacities (*q*_max_).

**Table:** Comparison of adsorption capacities related to AB1 and amino acid-Fe_3_O_4_ adsorbents

Adsorbent type	Dye used	Adsorption capacity (mg g^−1^)	Reference
Biopolymer-FeS NPs	Acid Black 1	20	10
Activated carbon (scrap tires)	Acid Black 1	14.5	44
Polyaniline/iron oxide	Acid Black 1	56	45
*Gracilaria persica* biomass	Acid Black 1	9	11
Unburned carbon	Acid Black 1	325	43
l-Arginine-Fe_3_O_4_ MNPs	Reactive Blue 19	125	4
Fe_3_O_4_@GPTMS@glycine MNPs	Acid Red 18	45	23
	Orange 1	49	
	Methylene blue	123	
	Methyl blue	158	
Fe_3_O_4_@GPTMS@lysine MNPs	Methylene blue	141	7
	Methyl blue	185	
l-Serine-Fe_3_O_4_ MNPs	Rhodamine B	7	17
l-Histidine-Fe_3_O_4_ MNPs	Acid Black 1	166.7	This work

Table 5 also compares the adsorption capacities of different amino acid-coated Fe_3_O_4_ nanoadsorbents for various dyes. In general, the *q*_max_ for histidine-Fe_3_O_4_ used is higher as compared to reported adsorbents, except unburned carbon for Acid Black 1 (ref. 43) and Fe_3_O_4_@GPTMS@lysine MNPs for methyl blue.^7^ Hence, the presence of surface-bound l-His plays a dominant role in enhancing the adsorption capacity of the adsorbent for AB1. Furthermore, the His-MNPs are superior to other adsorbents in terms of simple synthesis, easy separation, reusability and green/eco-friendly nature.

### Adsorption kinetics

Kinetics studies were conducted to investigate the controlling mechanism and rate of AB1 adsorption on His-MNPs. Kinetics data were collected at six different temperatures (30–80 °C) by analyzing the samples withdrawn at 3 min intervals until residual dye concentrations became constant. The initial concentration of dye, pH and adsorbent dosage were kept constant at 6.3 mg L^−1^, 4 and 3.3 g L^−1^, respectively. Four different models were applied for kinetics evaluations: pseudo-first order, pseudo-second order, intraparticle diffusion and the Boyd model. Table 6 presents the parameters of these kinetics models.

**Table:** Kinetics parameters for the adsorption of AB1 onto His-MNPs^a^

Kinetics parameters	30 °C	40 °C	50 °C	60 °C	70 °C	80 °C
**Experimental**						
*q*_e_	1.738	1.683	1.637	1.603	1.543	1.493

**Pseudo-first order model**						
*q*_e_ (mg g^−1^)	0.753	0.644	0.764	0.719	0.974	0.990
*k*_1_ (min^−1^)	0.190	0.188	0.228	0.221	0.285	0.370
*R* ^2^	0.999	0.998	0.999	0.998	0.977	0.986

**Pseudo-second order model**						
*q*_e_ (mg g^−1^)	1.822	1.755	1.720	1.686	1.627	1.572
*k*_2_ (g mg^−1^ min^−1^)	0.471	0.535	0.538	0.542	0.586	0.631
*R* ^2^	0.999	0.999	0.999	0.999	0.999	0.999

**Intraparticle diffusion model**						
*k*_id_ (mg g^−1^ min^−1/2^)	0.133	0.123	0.123	0.120	0.114	0.107
*C* (mg g^−1^)	1.164	1.152	1.118	1.097	1.070	1.050
*R* ^2^	0.921	0.899	0.893	0.904	0.892	0.893

**Boyd model**						
*R* ^2^	0.999	0.998	0.999	0.998	0.981	0.986

**Arrhenius parameters**						
*E*_a_ (kJ mol^−1^)	4.447					
*R* ^2^	0.893					

a Experimental conditions: pH = 4, initial AB1 concentration = 6.3 mg L^−1^, adsorbent dosage = 3.3 g L^−1^.

#### Pseudo-first-order and pseudo-second-order models

The linearized integral forms of the pseudo-first-order (Lagergren's) model and pseudo-second-order model of adsorption kinetics are given by eqn (1) and (2), respectively.^6,39^

1 ln(*q*_e_ − *q*_t_) = ln *q*_e_ − *k*_1_*t*

2 *t*/*q*_t_ = 1/(*k*_2_*q*_e_^2^) + *t*/*q*_e_

**Fig. 10:**
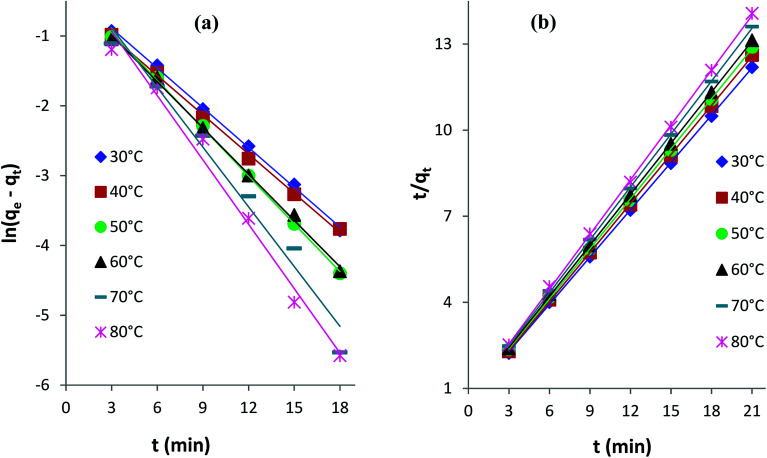
(a) Pseudo-first-order and (b) pseudo-second-order kinetics plots for the adsorption of AB1 dye onto His-MNPs at different temperatures.

*q*_e_ and *q*_t_ are the amounts of AB1 dye adsorbed (mg g^−1^) onto His-MNPs at equilibrium and at any time *t* (min), respectively. *k*_1_ is the rate constant of pseudo-first-order adsorption (min^−1^), and *k*_2_ is the rate constant of pseudo-second-order adsorption (g mg^−1^ min^−1^). The values of rate constants (*k*_1_ and *k*_2_), equilibrium adsorption capacity (*q*_e_) and correlation coefficients (*R*^2^) at different temperatures (listed in Table 6) were computed from linear plots of *t*/*q*_t_*versus t* for the pseudo-second-order model and ln(*q*_e_ − *q*_t_) *versus t* for the pseudo-first-order model (Fig. 10).

Table 6 indicates good compliance of AB1 adsorption on His-MNPs with pseudo-second-order kinetics as *R*^2^ > 0.999 and the *q*_e_ values from pseudo-second order plots are highly consistent and close to experimental *q*_e_ data, as compared to the respective pseudo-first-order parameters. The pseudo-second-order rate constants clearly show a positive relationship with temperature. The higher the temperature of the solution, the faster the collision of sorbent-sorbate molecules is and hence the AB1 molecules could bind to active sites on the nanoadsorbent surface with higher rates. Unlike *k*_2_, the experimental and calculated *q*_e_ (pseudo-second-order) values tend to be reduced at higher temperatures. This may be linked to the weakening of the bond between dye molecules and His-MNPs and hence greater possibility of bond breaking at higher temperatures.^34^

#### Intraparticle diffusion kinetics

The following eqn (3) expresses the intraparticle diffusion model:^4,35^

3 *q*_t_ = *k*_id_*t*^1/2^ + *C*

**Fig. 11:**
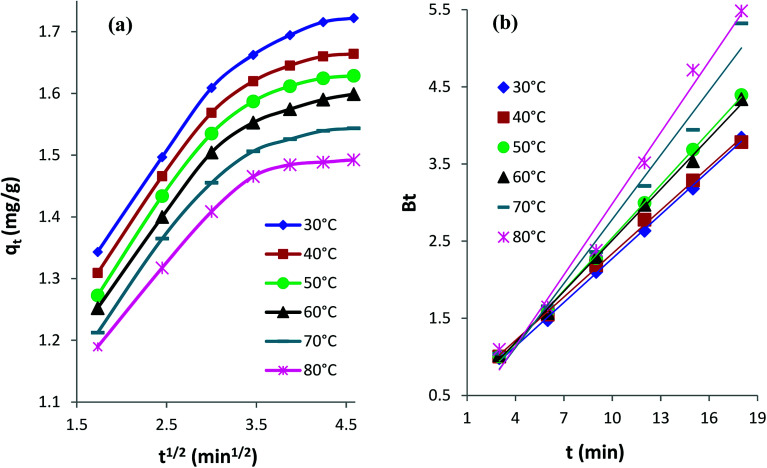
(a) Intraparticle diffusion and (b) Boyd kinetics plots for the adsorption of AB1 dye onto His-MNPs at different temperatures.

*k*_id_ is the rate constant (mg g^−1^ min^−1/2^) of intraparticle diffusion, and *C* reflects the thickness of the boundary layer. The *k*_id_ values for AB1 adsorption onto His-MNPs (Table 6) were computed from the slope of the linear plot of *q*_t_*versus t*^1/2^ at different temperatures (Fig. 11a).

The diffusion plots of *q*_t_*versus t*^1/2^ for AB1 adsorption (Fig. 11a) have two separate linear regions, indicating that the AB1 adsorption process follows two main steps. The initial linear portion can be assigned to the film or boundary layer diffusion of dye molecules, also called external mass transfer effect. The second linear portion can be attributed to the intraparticle diffusion effect. The intraparticle diffusion plots of AB1 at different temperatures showed linearity (*R*^2^ > 0.89), but none of the lines passed through the origin (*C* ≥ 1.05 mg g^−1^). This suggests that the adsorption of AB1 not only follows the intraparticle diffusion but the boundary layer diffusion,^39^ or some other mechanism such as ion-exchange,^37^ may also play a significant role in controlling the rate of AB1 adsorption onto His-MNPs. Furthermore, the magnitudes of both *k*_id_ and *C* decrease from 0.133 to 0.107 mg g^−1^ min^−1/2^ and from 1.164 to 1.050 mg g^−1^, respectively, on increasing the temperature from 30 to 80 °C. It is likely that fewer dye molecules are available for pore diffusion in the solid at higher temperatures, probably due to weak adsorbent–adsorbate linkages.

#### Boyd kinetic model

The Boyd model was employed to predict the main rate-limiting step for the uptake of AB1 by His-MNPs. The time (*t*) in minutes is plotted *versus B*_t_ in the Boyd plot; *B*_t_ is calculated from eqn (4) and (5).^46^

4 *B*_t_ = −0.4977 − ln(1 − *F*)

5 *F* = *q*_t_/*q*_e_

where *F* is the fraction of AB1 adsorbed at any time *t* (min), *q*_t_ is the amount of AB1 adsorbed (mg g^−1^) at time (*t*), and *q*_e_ is the amount of AB1 adsorbed (mg g^−1^) at equilibrium.

The Boyd plots for AB1 adsorption at different temperatures are depicted in Fig. 11b, and the corresponding correlation coefficients (*R*^2^) are provided in Table 6. It is suggested from Boyd plots that the process of AB1 adsorption is mainly governed by film diffusion, or the predominant rate-controlling mechanism for AB1 removal is the external mass transfer, based on the observations that Boyd plots are linear (*R*^2^ > 0.98) and they do not pass through the origin.^39^

#### Estimation of the activation energy of adsorption

Since the pseudo-second-order model is the best-identified model applied to AB1 dye adsorption onto His-MNPs, the pseudo-second-order rate constants of AB1 adsorption obtained at six different temperatures (Table 6) were used to compute the activation energy of the adsorption process for AB1 using the Arrhenius eqn (6).^47^

**Fig. 12:**
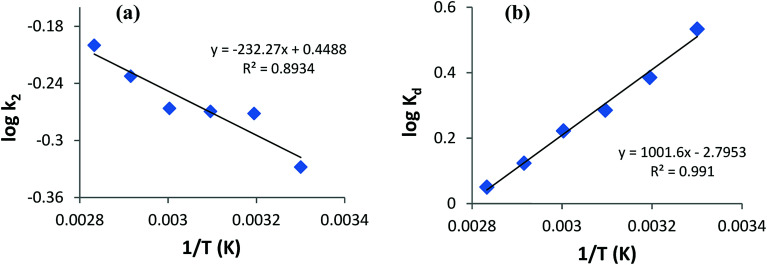
(a) Arrhenius plot for the activation energy and (b) Van't Hoff regression (thermodynamics) of adsorption of AB1 dye onto His-MNPs.

6 log *k*_2_ = log *A* – *E*_a_/(2.303*RT*)

where *k*_2_ is the rate constant of the pseudo-second-order adsorption (g mg^−1^ min^−1^), *E*_a_ is the Arrhenius activation energy of adsorption (kJ mol^−1^), *A* is a temperature-independent factor called the Arrhenius factor, *T* is the absolute temperature (K) of the solution, and *R* is the universal gas constant (8.314 J mol^−1^ K^−1^). To calculate *E*_a_, log *k*_2_ was plotted against 1/*T* (Fig. 12a) at constant pH (4), initial dye concentration (6.3 mg L^−1^) and adsorbent dosage (3.3 g L^−1^). The slope of this plot yields *E*_a_. The calculated *E*_a_ for AB1 adsorption is provided in Table 6.

The amount of activation energy could predict whether the adsorption type is mainly chemical or physical. The activation energy of chemisorption is normally more than 8 kJ mol^−1^, while it ranges from 1 to 8 kJ mol^−1^ for physisorption.^33^ The small calculated value of *E*_a_ for the current adsorption system (4.447 kJ mol^−1^) advocates that the adsorption of anionic dye AB1 onto His-MNPs is predominantly a physisorption process.

### Adsorption thermodynamics

Thermodynamics studies were executed to acquire better insight into the effect of temperature on AB1 adsorptive removal by magnetic NPs and to estimate the related thermodynamic parameters (Δ*H*°, Δ*S*° and Δ*G*°) shown in Table 7. To evaluate adsorption thermodynamics, equilibrium adsorption data were collected at six different temperatures (303, 313, 323, 333, 343 and 353 K) using 6.3 mg L^−1^ AB1 and 3.3 g L^−1^ His-MNPs at pH 4. The variation in the entropy (Δ*S*°) and enthalpy (Δ*H*°) were computed from the intercept and slope of the plot of log *K*_d_*versus* 1/*T*, respectively (Fig. 12b), using the Van't Hoff eqn (7):^36,41^

7 log *K*_d_ = (Δ*S*°/2.303*R*) − (Δ*H*°/2.303*RT*)

8 *K*_d_ = *q*_e_/*C*_e_

where *K*_d_ is the distribution coefficient of adsorption (L g^−1^) calculated from the AB1 amount adsorbed onto His-MNPs at equilibrium (*q*_e_, mg g^−1^) and AB1 concentration at equilibrium in the liquid phase *C*_e_, mg L^−1^. *T* is the absolute temperature (K), and *R* is the universal gas constant (8.314 J mol^−1^ K^−1^).

**Table:** Thermodynamics parameters of AB1 adsorption onto His-MNPs

*T* (K)	*K*_d_ (L g^−1^)	Δ*G*° from eqn (9) (kJ mol^−1^)	Δ*G*° from eqn (10) (kJ mol^−1^)	Δ*H*° (kJ mol^−1^)	Δ*S*° (J mol^−1^ K^−1^)	*R* ^2^
303	3.415	–2.960	–3.094	–19.18	–53.52	0.991
313	2.430	–2.425	–2.311			
323	1.930	–1.890	–1.767			
333	1.668	–1.355	–1.416			
343	1.329	–0.819	–0.811			
353	1.123	–0.284	–0.340			

The change in the standard Gibbs free energy (Δ*G*°) during adsorption at various temperatures was estimated from the following equations:^40^

9 Δ*G*° = Δ*H*° − *T*Δ*S*°

10 Δ*G*° = −*RT* ln *K*_d_

Based on negative Δ*G*° values (−3.094 to −0.284 kJ mol^−1^) at various temperatures (30 to 80 °C), the adsorption of AB1 onto His-MNPs is spontaneous and thermodynamically favorable.^9^ Thermodynamic feasibility and the degree of spontaneity of the adsorption process decreases on increasing the temperature as suggested by higher Δ*G*° values at higher temperatures. The negative Δ*H*° value (−19.18 kJ mol^−1^) reflects the exothermic behavior of AB1 adsorption. This is in agreement with the lowering of the equilibrium adsorption capacity with mounting temperature, which may be due to weak attractive forces between sorbent/sorbate molecules. The negative Δ*S*° value (−53.52 J mol^−1^ K^−1^) corresponds to reduced randomness at the solid–liquid interface during AB1 adsorption onto the nanoadsorbent. This is attributed to minor structural changes in the adsorbent and adsorbate.^37^ The values of the Gibbs free energy changes and enthalpy changes may be used to identify the adsorption process as physical or chemical sorption as follows:

**Table:** 

	Physical adsorption	Chemical adsorption	Reference
Δ*G*° range	0 to −20 kJ mol^−1^	−80 to −400 kJ mol^−1^	48
Δ*H*° range	2.1 to 20.9 kJ mol^−1^	80 to 200 kJ mol^−1^	41

The small magnitudes of Δ*G*° and Δ*H*° observed in this study indicate that the uptake of AB1 onto His-MNPs is predominantly a physical adsorption process.

### Recycling of His-MNPs

The regeneration of the adsorbent after loading and its reuse make the adsorption application more economical. The recovery of AB1 from used adsorbent was checked by desorption studies in three different solutions/eluents (1 M HCl, 1 M NaOH and 9 : 1 (v/v) CH_3_OH : CH_3_COOH mixture) to find the best desorbing medium, and results are shown in Table 8.

**Table:** Effect of different eluents on AB1 desorption from used His-MNPs^a^

S. no.	Eluent	Desorption%	Desorption equilibrium time (min)
1	HCl (1 M)	0.0	—
2	NaOH (1 M)	84.6	55
3	CH_3_OH : CH_3_COOH (9 : 1)	2.2	15

a Experimental conditions: adsorbent dosage = 3.3 g L^−1^, temp. = 30 °C, contact time = 60 min.

The highest percent desorption of AB1 (84.58%) from used His-MNPs (3.3 g L^−1^) was achieved by 1 M NaOH with the obvious blue color of the dye in the solution. This is because the positive charge on the cationic His-MNPs surface may get neutralized in highly basic solution, making electrostatic attraction between His-MNPs and anionic dyes less favorable, thus facilitating desorption of AB1 in basic solution. The 1 M HCl solution remained unsuitable for desorbing AB1 dye; rather, it showed some decomposition of His-MNPs as indicated by the slight yellow color of ferric ions in the solution. The effective desorption of AB1 by His-MNPs in basic medium as compared to no desorption in acidic medium agrees well with the results of the pH study (Fig. 7a). Therefore, we selected NaOH solution for further reusability studies. Zhang *et al.* (2014) also observed significant desorption of anionic dyes from lysine coated MNPs in NaOH due to similar electrostatic adsorbent–adsorbate interactions.^7^ However, Ge *et al.* (2012) found 0.1 M HCl to be better for desorbing different heavy metal ions from Fe_3_O_4_@APS@AA-*co*-CA MNPs, while there was corrosion of adsorbent at higher H^+^ concentration. The small desorption (2.16%) in an organic solvent mixture (9 : 1 CH_3_OH : CH_3_COOH) may be attributed to some dissolution of AB1 (organic ionic dye) by polar organic solvents.^33^

**Fig. 13:**
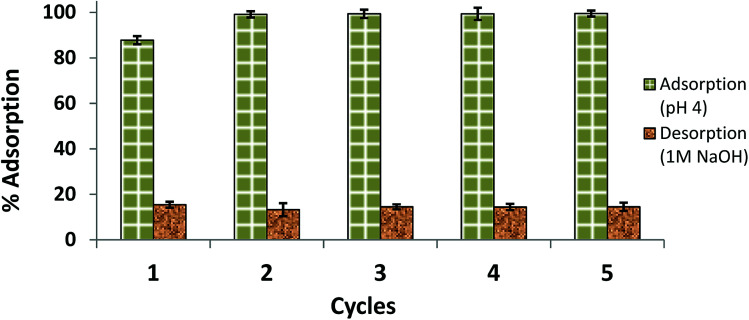
Reusability of His-MNPs for 5 cycles (initial AB1 concentration = 6.3 mg L^−1^, adsorbent dosage = 3.3 g L^−1^, temp. = 30 °C, contact time = 60 min).

The results of the reusability test are illustrated in Fig. 13 as five consecutive adsorption/desorption cycles. Compared to the AB1 removal efficiency in the first cycle (87.80%), the adsorption efficiency was significantly enhanced (99.13–99.50%) in the next four cycles. This may be due to modification of the surface and internal structure of the adsorbent after the first cycle; the adsorbent may become softer with more surface area available on it after interaction with NaOH and multiple washings. The percent desorption remained almost the same for 5 cycles (84.58–85.50%). Hence, a part of the dye (about 14–16%) remained adsorbed, which may be due to van der Waals attraction and hydrogen bond formation.^49^ Another interesting observation was that the equilibrium time of desorption (55 min) observed in the first cycle was decreased in successive cycles, reaching 25 min for the next four desorptions; this may also be because of changes in the surface and internal structure of the adsorbent. Therefore, His-MNPs is a potential adsorbent for anionic dyes, which can be easily regenerated and has good reusability.

### Mechanism of removal of AB1 by His-MNPs

**Fig. 14:**
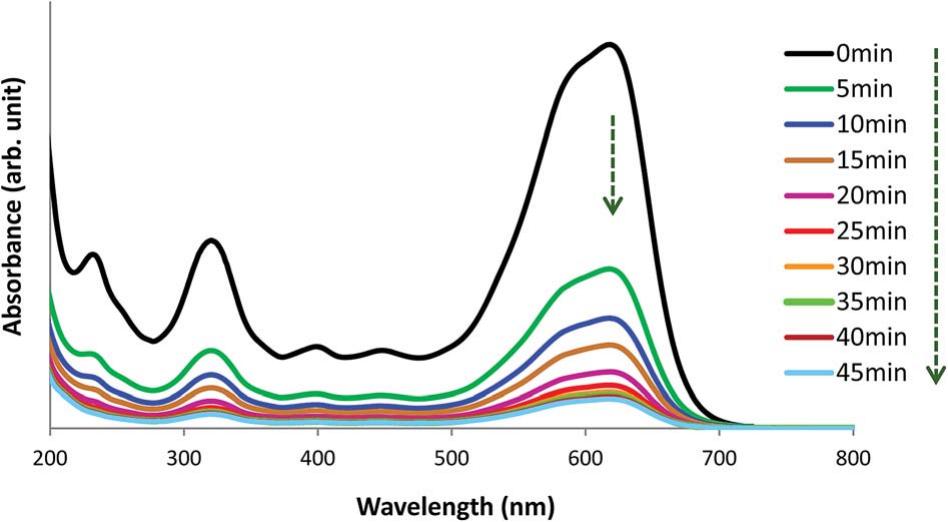
UV-visible spectral view of the removal of AB1 with His-MNPs(s) at various time intervals (pH = 4, initial AB1 concentration = 24.7 mg L^−1^, adsorbent dosage = 0.2 g L^−1^, temp. = 30 °C).

The UV-visible spectrum of pure AB1 dye (Fig. 14, 0 min) illustrates two characteristic intense absorption bands at 618 nm (*λ*_max_) and 321 nm, attributed to the dye chromophore (–N=N–) and aromatic ring structure, respectively.^8^Fig. 14 shows that when an aqueous solution of AB1 is treated with His-MNPs under certain conditions (pH 4; AB1 concentration of 24.7 mg L^−1^; NPs amount, 0.2 g L^−1^; temperature, 30 °C), the intensity of all the bands is reduced without affecting the position or shape of any band. The decrease in band intensity indicates the removal of AB1, which continues in similar trend with the passage of time until maximum dye removal or equilibrium is achieved (92.37% removal in 45 min, calculated at *λ*_max_). In the absence of any light irradiation or induced oxidant, the phenomenon of AB1 adsorption rather than its degradation could be proposed as a dye removal mechanism for the current study. Additionally, no destruction in the UV-visible spectrum of AB1 (except reduction of band intensity) upon addition of His-MNPs also suggests the adsorption mechanism and absence of AB1 degradation. This viewpoint is justified by the study of Abdullah and Kou (2015),^50^ and Mohammadi *et al.* (2016);^51^ they observed significant destruction in the UV-visible spectrum of AB1 due to its photocatalytic degradation. The adsorption of AB1 was also confirmed when used His-MNPs released AB1 color in tested eluents (aq. NaOH and methanol-acetic acid mixture) due to desorption, and the resulting eluates exhibited characteristic absorption bands for AB1 in the UV and visible region.

**Fig. 15:**
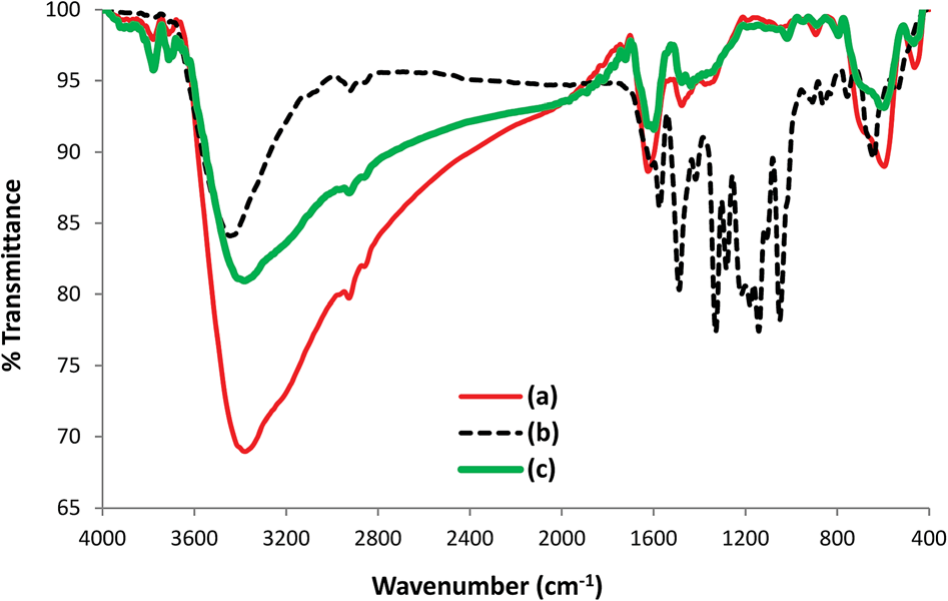
FT-IR spectra of (a) untreated His-MNPs, (b) AB1 dye and (c) dye-treated His-MNPs.

**Scheme 2:**
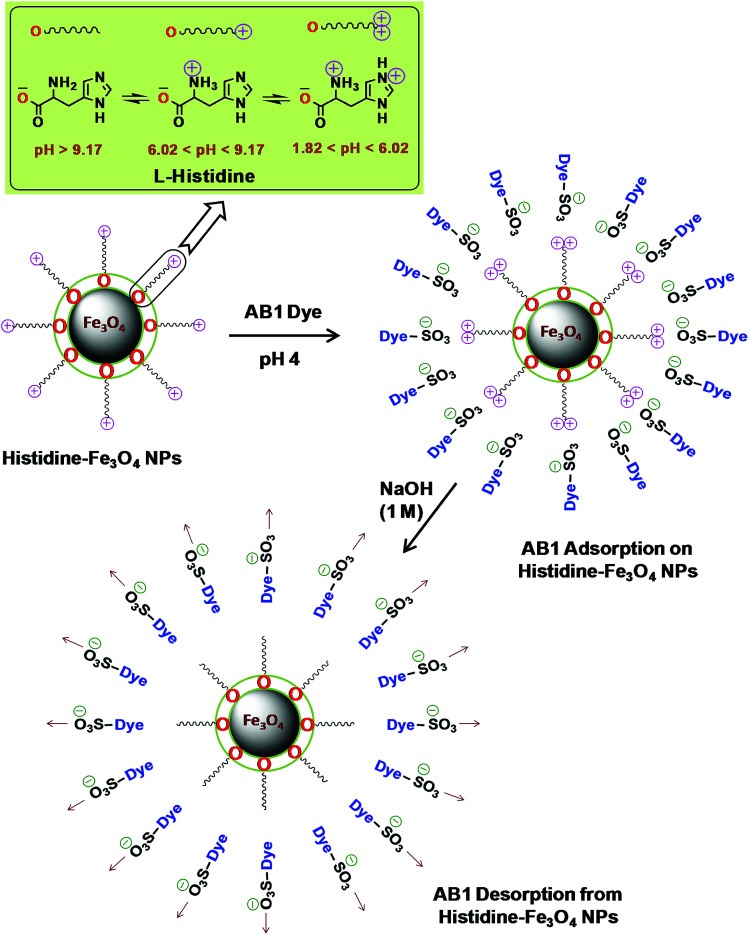
Possible adsorption/desorption mechanism of His-MNPs for AB1.

To further investigate the nature of the interaction of AB1 with His-MNPs, the FT-IR spectra of untreated His-MNPs (Fig. 15a), pure AB1 dye (Fig. 15b) and AB1-treated His-MNPs (Fig. 15c) were compared, which provided valuable mechanistic clues. The intensity of the band at 3381 cm^−1^, referring to NH_2_ stretching in His-MNPs, is significantly reduced (15%) with shifting of the band to 3379 cm^−1^ after treatment with AB1. This shows the adsorption of AB1 and that the amino group on the surface of His-MNPs strongly interacts with AB1. Similar changes were observed by Kousha *et al.* (2012) due to the adsorption of AB1 on algal biomass.^11^ The appearance of a new sharp peak at 1598 cm^−1^ for dye-treated His-MNPs corresponds to –N=N– stretching of loaded AB1,^52^ showing that AB1 is adsorbed without cleavage of the azo bond. Three more new peaks related to adsorbed AB1 were observed at 1437, 1019 and 890 cm^−1^, which were assigned to –NO_2_ stretching, C–O stretching of alcohol and C–H deformation of the tri-substituted benzene ring,^52^ further confirming the adsorption of AB1 on NPs. The strong band at 1142 cm^−1^ appeared due to the S=O stretching vibration of SO_3_Na on the aromatic ring in pure AB1,^10^ which was not observed in the FT-IR spectrum of AB1 loaded His-MNPs. This may be due to the strong electrostatic interactions of the sulfonate (–SO_3_^–^) groups of anionic AB1 with positive His-MNPs, probably through an amino group. Some other strong absorption bands in the region of 1330–1219 cm^−1^ for dye alone, attributed to the C–N stretching of aromatic carbons attached to nitrogen functionalities,^53^ were also absent in the case of AB1-treated His-MNPs because of the adsorption of dye onto the adsorbent surface. The observation that lower (acidic) pH facilitates AB1 removal by His-MNPs compared to the basic environment also establishes the existence of ionic interactions between AB1 and His-MNPs. Based on all the above findings, the probable mechanism of interaction between AB1 and His-MNPs during adsorption and desorption is depicted in Scheme 2.

l-Histidine, an amino acid, is amphiprotic in nature as it changes its surface charge with pH, having five different pH-dependent ionic forms. Scheme 2 illustrates three different ionic forms of free l-histidine that predominantly exist at three different pH ranges: anionic form (–COO^–^, NH_2_) at pH > 9.17, zwitterionic form (–COO^–^, NH_3_^+^) at 6.02 < pH < 9.17, and cationic form (–COO^–^, NH_3_^+^, =NH^+^–) at 1.82 < pH < 6.02.^54^ Since the synthesis of His-MNPs was carried out in basic environment (pH 9), the negatively charged carboxylate oxygen (strong nucleophile) of l-histidine was expected to bind through unsaturated Fe^3+^/Fe^2+^ found on the Fe_3_O_4_ NPs surface, yielding a positive charge on the His-MNPs surface due to protonated amino (–NH_3_^+^) groups. In the acidic environment (pH = 1.8–6.02), the pyrrole imino group of l-histidine also becomes protonated (=NH^+^–). Due to the presence of a large number of cationic sites on His-MNPs, their electrostatic interactions with the anionic sulfonate groups of AB1 (anionic dye) increase at lower pH, which justifies the increase in AB1 adsorption onto His-MNPs with a decrease in pH. As the pH increases from 6.02, the positive charge on the amino and pyrrole imino groups of l-histidine starts to be neutralized. This decreases the electrostatic attraction between the dye and His-MNPs, causing a decrease in adsorption at higher pH. The present finding of NaOH as the best desorption solvent for AB1 as compared to HCl and organic solvents is also in accord with the suggested mechanism of interaction between AB1 and His-MNPs. Xu *et al.* (2018) also observed similar pH-responsive behavior of Chitosan/Fe_3_O_4_ NP towards anionic dyes.^1^

Some equilibrium, kinetics and thermodynamics parameters (D–R free adsorption energy, *E*_a_, Δ*G*° and Δ*H*°) estimated from the present adsorption data strongly advocate that the nature of adsorption is physisorption. There are a number of published reports that describe the electrostatic adsorbent–adsorbate interactions (proven by pH and ZPC studies) as physisorption based on small estimated adsorption related energies (*E*_D–R_, *E*_a_, Δ*H*, Δ*G*, *etc.*),^35,41,55–57^ while many others describe similar electrostatic interactions as chemisorption because of higher adsorption energies within the chemisorption reference range.^17,33,36,58,59^ Some researchers interpret electrostatic adsorptive interactions verified by pH as only a chemical affinity, even though their estimated adsorption energies are lower and not consistent with referenced chemisorption energies.^4,9,60^ Furthermore, monolayer and multilayer adsorption energies for a single adsorbent–adsorbate case can be affected by experimental conditions (temperature, pH, *etc.*) as shown by Yu *et al.* (2001); they interpreted the simultaneous mechanism as being ‘physisorption enhanced by chemical effect’ for Reactive Blue 19 onto the functionalized resin.^61^ Inyinbor *et al.* (2016) also deduced the adsorption of rhodamine B onto *Raphia hookeri* fruit epicarp as being a combination of physical and chemical effects, although their estimated *E*_D–R_, Δ*H* and Δ*G* values are consistent with physisorption.^62^ Therefore, detailed research is recommended to reset the boundary limits of adsorption-related energies (or at least these must be given with certain experimental conditions for reference) with respect to chemisorption or physisorption or a new third type of electrostatic adsorption to avoid ambiguity in interpretation. The present study identifies the crucial role of His-MNPs as the nanoadsorbent for the removal of AB1 that would be used in the future on real textile effluents to remove hazardous dyes. The dye removal efficiency could be further enhanced by finding the additional role of His-MNPs as a photocatalyst.

## Conclusions

Green magnetic Fe_3_O_4_ nanoparticles (MNPs) functionalized with biocompatible l-histidine (l-His) were successfully synthesized and characterized. Applying His-MNPs as the adsorbent, the removal efficiency of AB1 was critically influenced by pH, temperature, dye concentration, adsorbent amount and pre-soaking of adsorbent in an organic environment. Due to the availability of positive charge on His-MNPs in acidic medium rendered by an amino group and the imidazole ring nitrogen of l-histidine, His-MNPs can serve as a promising adsorbent for the removal of anionic dyes from aqueous solutions through electrostatic interactions, which is well corroborated by pH, IR and desorption studies. The His-MNPs may also be effective against cationic dyes in basic medium, which should be verified by further studies. Thermodynamic parameters (Δ*G*° and Δ*H*°), *E*_a_, and UV-visible spectra all validate that the removal of AB1 by His-MNPs occurs through physisorption. The His-MNPs were superior to other adsorbents due to facile synthesis, pH-responsiveness, high adsorption capacity, easy magnetic separation, their environmentally friendly nature and reusability. His-MNPs can provide an efficient, economic and sustainable approach to remove azo dyes from water. The His-MNPs should be explored further as a potential adsorbent for the treatment of real textile or other industrial effluents.

## Conflicts of interest

There are no conflicts to declare.

## Supplementary Material

RA-009-C8RA09279F-s001
