# Supplementary material for: Convenient pH-responsive removal of Acid Black 1 by green l-histidine/iron oxide magnetic nanoadsorbent from water: performance and mechanistic studies
Source: RSC Adv. 2019 Jan 23;9(6):2978–96. doi: 10.1039/c8ra09279f (PMC9059959; doi:10.1039/c8ra09279f)
Supplement: RA-009-C8RA09279F-s001 [file RA-009-C8RA09279F-s001.pdf]

Electronic Supplementary Material (ESI) for RSC Advances.
This journal is © The Royal Society of Chemistry 2019

RSC Advances

ELECTRONIC SUPPLEMENTARY INFORMATION

Convenient pH-Responsive Removal of Acid Black 1 by Green L-Histidine/Iron Oxide Magnetic Nanoadsorbent from Water: Performance and Mechanistic Studies

Qurrat-ul-Ain,[a]* Jaweria Khatoon,[b] Muhammad Raza Shah,[c] Muhammad Imran Malik,[c] Iffat Abdul Tawab Khan,[b] Sumaira Khurshid[b] and Raheela Naz[a]

[a] *Department of Chemistry, Faculty of Science, University of Karachi, Karachi-75270, Pakistan*
Email: qurrat_chem@uok.edu.pk; Fax: +92.21.99261330; Tel: +92.21.99261300
[b] *Department of Chemistry, Federal Urdu University of Arts, Science and Technology, Gulshan-e-Iqbal Campus, Karachi-75300, Pakistan*
[c] *H. E. J. Research Institute of Chemistry, ICCBS, University of Karachi, Karachi 75270, Pakistan*
Email: raza.shah@iccs.edu

Effect of Temperature on AB1 Adsorption: The effect on AB1 adsorption by Histidine-functionalized magnetic nanoparticles (His-MNPs) was determined as a function of temperature using six different temperatures (30, 40, 50, 60, 70 and 80ºC), keeping other variables constant (pH 4, adsorbent 3.3 mg/L, dye 6.3 mg/L, equilibrium stirring time 50 min). Fig. S1 shows that AB1 adsorption on His-MNPs is gradually decreased with increase in temperature. The maximum dye uptake was observed at 30ºC. For every 10ºC rise in temperature, about 2–3% decrease in the percent dye removal and adsorption capacity was observed. The percent adsorption decreased from 91.92 to 78.92% while adsorption capacity decreased from 1.738 to 1.492 mg/g at equilibrium on varying the temperature from 30 to 80ºC. This reduction in adsorption ability on increase in temperature is mainly due to decreased surface activity, and the bond weakening between AB1 molecules and active sites of His-MNPs adsorbent owing to increased mobility and decreased viscosity at higher temperatures, suggesting exothermic AB1 adsorption.[1] Dye adsorption is considered as a replacement of water molecules by dye species, and the net enthalpy is a balance between desorption of water and adsorption of dye. High temperatures, affecting this balance, may enhance AB1 desorption rather than its adsorption by His-NPs. Mahto et al. (2014) also reported decrease in adsorption efficiency with increase of temperature for adsorption of Malachite Green by polyaniline-magnetite NPs.[2] In contrast, some authors experienced enhancement of adsorption efficiency of a magnetite adsorbent towards Rhodamine B at higher temperatures, implying an endothermic adsorption process.[3]

Effect of Concentration of Electrolyte on AB1 Adsorption: The influence of presence of electrolyte or ionic strength on the removal of AB1 by His-MNPs was investigated by using different concentrations of NaCl (0, 0.2, 0.4, 0.6 and 0.8 M) in dye aqueous solution at pH 4.0 and 6.2. Other experimental conditions were kept constant (temperature 30ºC, adsorbent amount 3.3 mg/L, adsorbate concentration 6.3 mg/L, equilibrium stirring time 50 min). The presence of NaCl as an electrolyte did not effectively influence the percent adsorption and adsorption capacity of His-MNPs for AB1 even at higher salt concentrations at pH 4.0 and 6.2 (Fig. S2). It can be suggested that the electrostatic attraction between cationic His-MNPs and anionic AB1 dye remains alive in the presence of salt, and this interaction is not influenced by external electrostatic forces, where salt ions may serve as ionic bridge between His-MNPs and dye molecules. However, the presence of salt can slightly reduce the rate of adsorption of AB1. This is based on the observation that equilibrium time of AB1 removal was increased from 25 to 45 minutes on varying NaCl concentration from 0 to 0.8 M (Fig. S6). This is probably due to steric hindrance caused by salt ions, which may impede direct electrostatic attraction between His-MNPs and dye molecules, leading to slower adsorption. Unlike our results, Dalvand et al. (2016) observed decrease in the percentage removal of anionic azo dye Reactive Blue 19 by L-arginine-functionalized Fe_3O_4 MNPs on increasing NaCl concentration.[4] The absence of any negative effect by electrolyte on adsorption capacity could be of great significance in the treatment of real textile effluents that are usually enriched with variety of salts being applied during dyeing process.

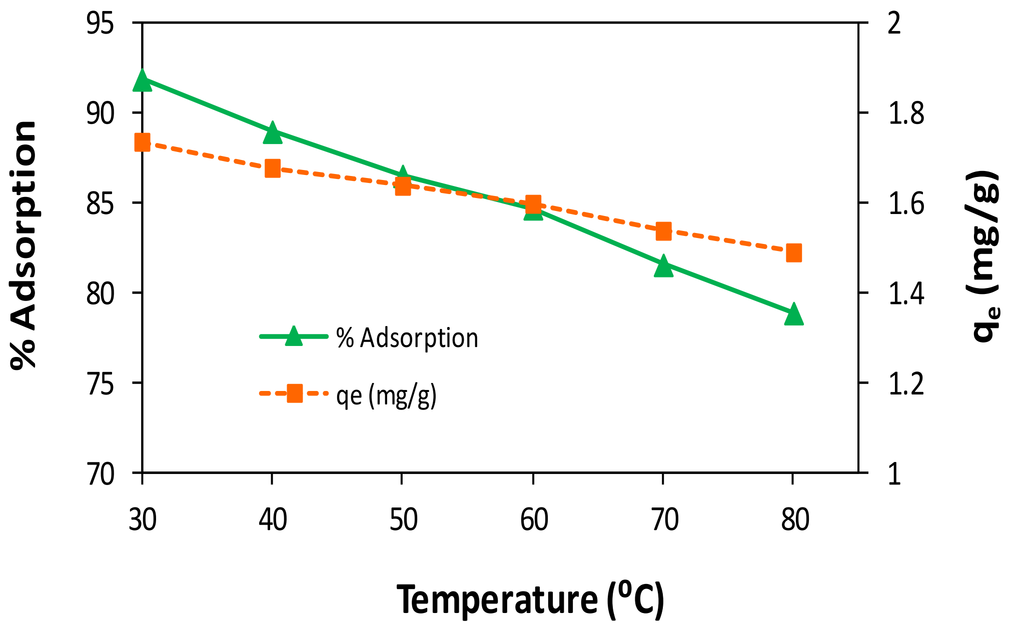

Fig. S1 Effect of temperature on adsorption of AB1 by His-MNPs

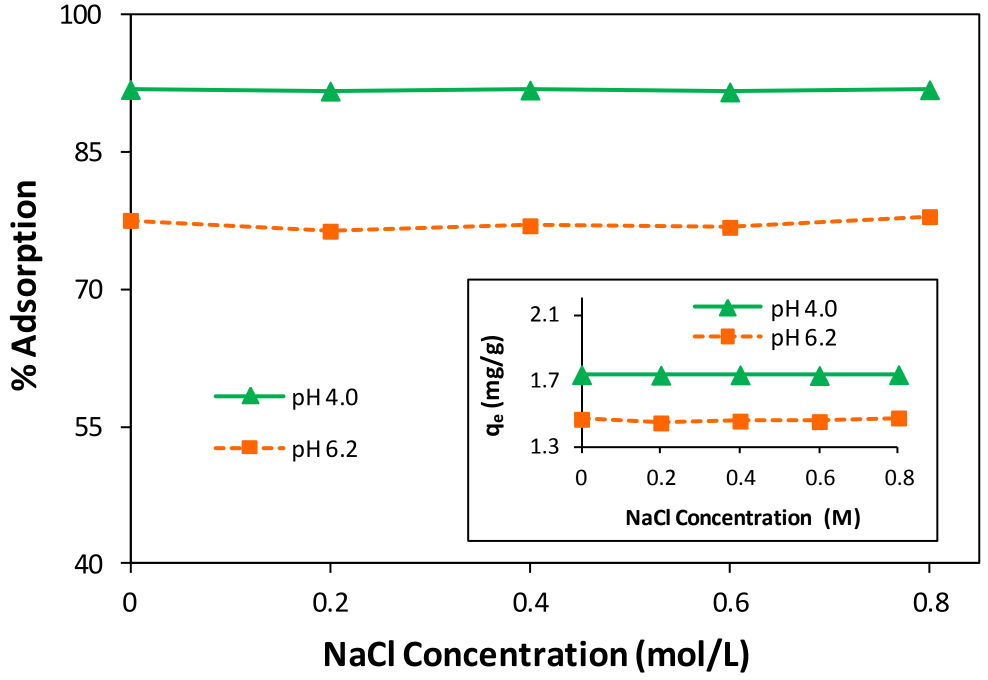

Fig. S2 Effect of electrolyte on adsorption of AB1 His-MNPs

Effect of His-MNPs Dosage on AB1 Adsorption: To investigate the impact of dosage of His-MNPs on AB1 adsorption, the quantity of His-MNPs was varied from 0.2 to 20 g/L at pH 4, dye concentration 6.3 mg/L and temperature 30°C. After 50 min equilibrium stirring time, the percent adsorption and adsorption capacity were calculated and their variation in relation to the His-MNPs dose is shown in Fig. S3. The AB1 removal percentage was ramped from 85.33 to 99.21% as the dose of His-MNPs raised from 0.2 to 20 g/L. This is primarily because of the availability of large surface area and hence more number of exposed active sites for binding of AB1 at higher His-MNPs amounts.[4] However, the difference in percent adsorption at higher adsorbent doses (13.3, 16.6 and 20 g/L) is very small (almost constant) compared to change in the percent removal at lower doses (0.2, 0.7, 3.3, 6.6 and 10 g/L), probably owing to overlapping of active adsorption sites caused by over-crowding of His-MNPs at their higher amounts. This idea is also strengthen by relatively slower adsorption equilibrium rates observed at higher adsorbent doses (e.g., 35 min equilibrium time for 20 g/L) compared to fast equilibrium attained at lower adsorbent doses (e.g., 15 min equilibrium time for 0.2 g/L) (Fig. S6). Contrary to % AB1 removal, the q_e was negatively affected by the increase of adsorbent dosage, which may be attributed to splitting effect of concentration gradient (flux) between adsorbent and asorbate.[5,6] It can be deduced from this study that equilibrium uptake of AB1 is strongly influenced by His-MNPs dose.

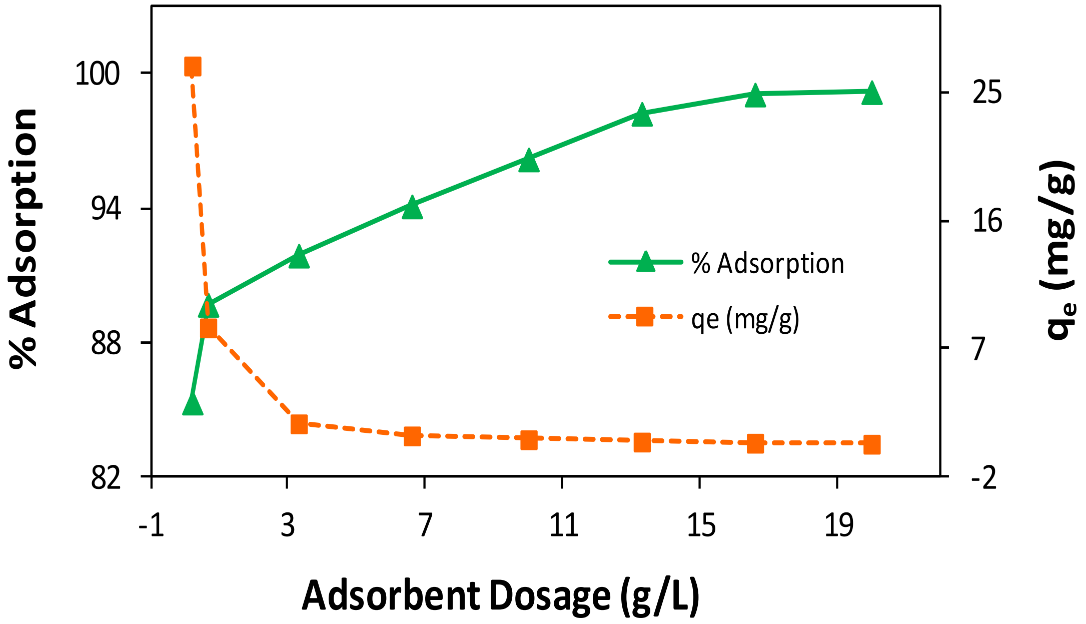

Fig. S3 Effect of adsorbent amount on adsorption of AB1 by His-MNPs

Effect of Initial Concentration of AB1 on its Adsorption: The initial AB1 concentration critically influenced its uptake by His-MNPs and His-MNPs(s). Fig. S4a illustrates the effect of AB1 concentration (over a range of 0.7 to 40 mg/L) on adsorption of His-MNPs (3.3 g/L) at pH 4 and pH 5, while Fig. S4b illustrates the influence on His-MNPs(s) (0.2 g/L) of varying ABI concentration (24.7 to 40 mg/L) at pH 4. The conditions of temperature and stirring time were kept constant at 30ºC and 50 min, respectively, during this study. Fig. S4a and b indicate that increasing the initial concentration of AB1 in the tested range decreases the percent removal of dye, e.g., from 97.82% to 83.21% by His-MNPs and from 92.37% to 73.27% by His-MNPs(s) at pH 4. It may be because of the availability of limited number of active sites on His-MNPs not ample to adsorb all AB1 molecules and saturation of active sites at higher dye concentrations.[7] However, the equilibrium adsorption capacity showed a positive trend with AB1 initial concentration, as shown by increase in the q_e value from 0.184 to 10.32 mg/g for His-MNPs (Fig. S4a) and from 106.0 to 152.5 mg/g for His-MNPs(s) (Fig. S4b) on increasing AB1 concentration. This may be observed due to more number of dye molecules available for the equilibrium and their interaction with His-MNPs.[8,9] Kakavandi et al. (2013) also observed similar trend of percent adsorption and adsorption capacity on changing adsorbate concentration.[7] The maximum experimental adsorption capacity of synthesized L-His coated magnetic nanoparticles against AB1 observed from this study was 152.5 mg/g that is higher than maximum q value of several previously reported magnetic nanoadsorbents, for example, $Fe_3O_4@SiO_2-NH_2$ NPs (84.75 mg/g against Acid Red 114),[10] Fe_3O_4@DAPF (99.60– 142.1 mg/g against Amaranth, Orange II and Acid Red 18),[11] Fe_3O_4/MgO NPs (37.98 mg/g against Amaranth),[12] and some other Fe_3O_4 adsorbents listed in Table 5.

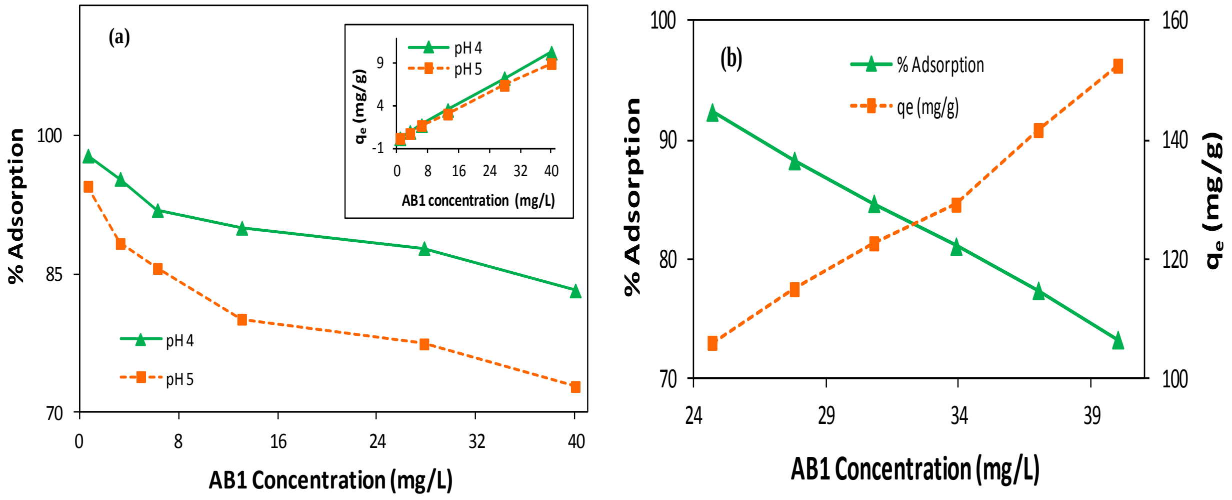

Fig. S4 Effect of AB1 (adsorbate) concentration on its adsorption by (a) His-MNPs at pH 4 and pH 5, and (b) His-MNPs(s) at pH 4

Effect of Contact Time on AB1 Adsorption: To study contact time between dye and adsorbent to reach adsorption equilibrium is crucial in designing cost-effective adsorbent and in predicting mechanism of dye removal. The large dye removal at short contact time clues that the main reaction controlling step is film diffusion; however, the large dye adsorption at long time intervals reveals the contribution of intraparticle diffusion in the dye removal process.[12] The effect of stirring time on the adsorption of AB1 by L-His coated MNPs was studied in all experiments (study of pH, temperature, electrolyte, adsorbent dosage and dye concentration) to investigate the required equilibrium time for adsorption. Fig. S5 shows the data of AB1 adsorption (% removal and q_t) as a function of contact time for different doses of His-MNPs (at 3.3, 6.6, 10.0 and 13.3 g/L His-MNPs). The relationship between contact time and adsorption capacity (q) for temperature study (30–80ºC) has been shown in kinetics section (Fig. 12 and 13). The variation in the UV-visible spectrum at various time intervals during AB1 removal by His-MNPs(s) for adsorbate concentration effect study (at 24.7 mg/L dye) is also shown in mechanism section (Fig. 17). It is clearly evident from given figures that adsorption starts immediately within 5 min, and most of the AB1 dye (about 90%) adsorbed within first 15 to 25 min of adsorption process during which the removal of dye enhanced with increasing the contact time. The initial high rate is probably because of greater surface area of His-MNPs being available for AB1 to adsorb at the beginning. The adsorptive removal of AB1 reached equilibrium within 45 minutes or less, after which the effect on dye removal percentage was insignificant in all cases due to saturation of adsorbent surface with AB1 (Fig. S5 and S6). The fast AB1 removal within short contact time predicts that the film diffusion (i.e., transfer of AB1 from aqueous phase to external His-MNPs surface) is a predominant step in the AB1 removal compared to intra-particle diffusion. Compared to reported iron oxide nanoadsorbents,[13,11,10] our His-MNPs exhibit fast adsorption ability against anionic dye AB1. It may be credited to larger surface area and effective ionic interaction offered by His-MNPs for AB1.

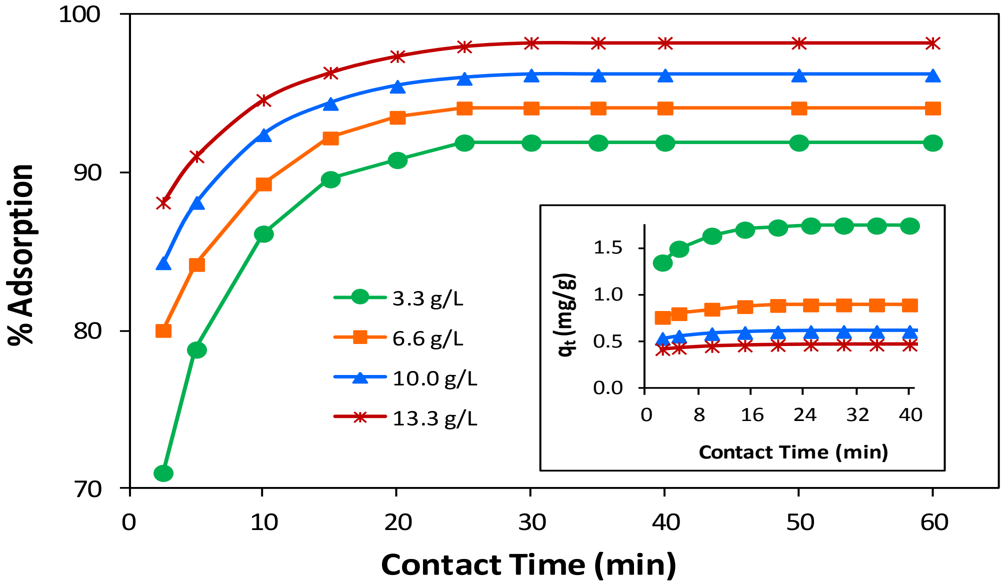

Fig. S5 Effect of contact/ stirring time on adsorption of AB1 by His-MNPs at different adsorbent dosages (pH = 4, initial AB1 concentration = 6.3 mg/L, adsorbent dosage = 3.3–13.3 g/L, temp. = 30°C)

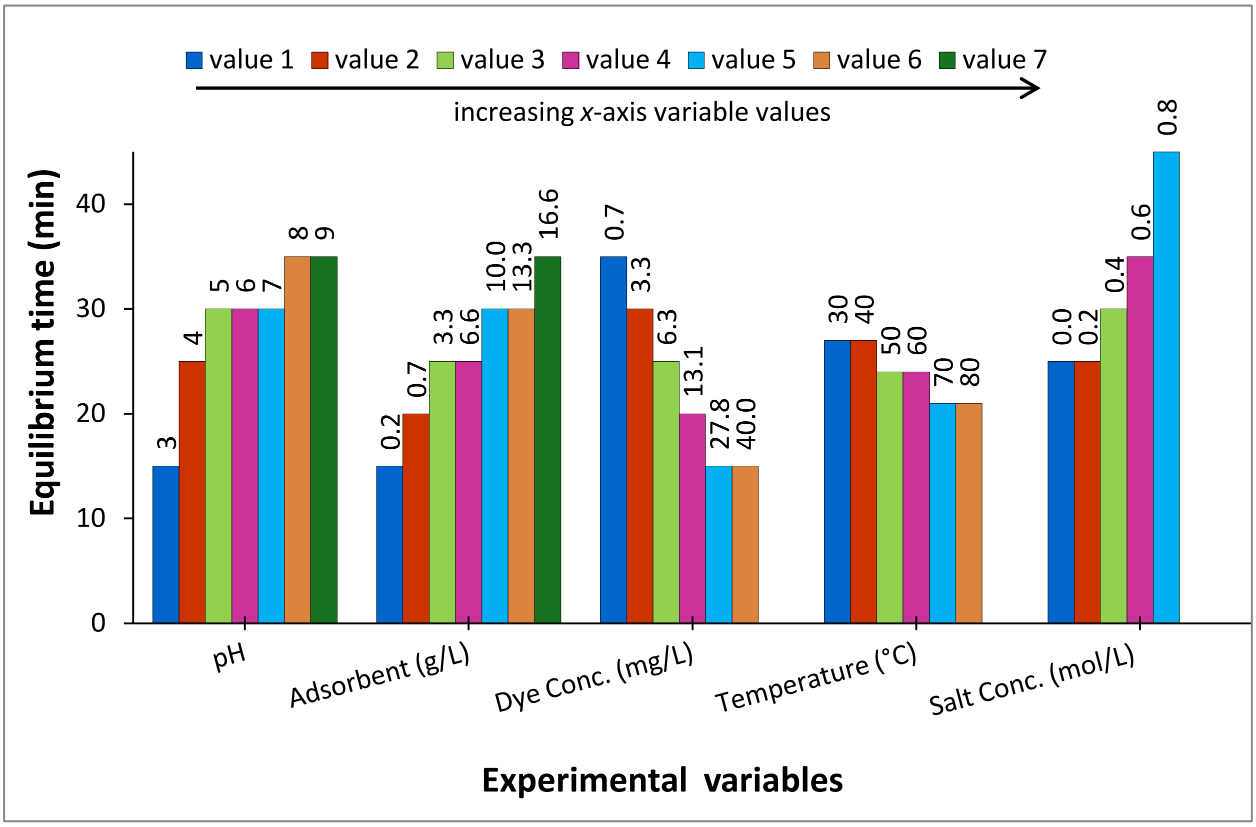

Fig. S6 Effect of experimental variables (pH, adsorbent dosage, dye concentration, temperature and NaCl salt concentration) on equilibrium time of AB1 adsorption by His-MNPs. Variable values are provided on top of respective bars (other variable conditions: pH = 4, initial AB1 concentration = 6.3 mg/L, adsorbent dosage = 3.3 g/L, temp. = 30°C)

References

1 A. Zargar, H. Parham and M. Rezazade, *J. Chin. Chem. Soc.*, 2011, **58**, 694–699.

2 T. K. Mahto, A. R. Chowdhuri and S. K. Sahu, *J. Appl. Polym. Sci.*, 2014, 131, 40840–40848.

3 N. Belachew, D. R. Devi and K. Basavaiah, *J. Exp. Nanosci.*, 2017, **12(1)**, 114–128.

4 A. Dalvand, R. Nabizadeh, M. R. Ganjali, M. Khoobi, S. Nazmara and A. H. Mahvi, *J. Magn. Magn. Mater.*, 2016, **404**, 179–189.

5 G. Vijayakumar, R. Tamilarasan and M. Dharmendirakumar, *J. Mater. Environ. Sci.*, 2012, **3(1)**, 157–170.

6 Y. S. Ho and A. E. Ofomaja, *J. Hazard. Mater.*, 2006, **B129**, 137–142.

7 B. Kakavandi, A. J. Jafari, R. Z. Kalantary, S. Nasseri, A. Ameri and A. Esrafily, *Iranian J. Environ. Health Sci. Eng.*, 2013, **10(1)**, 1–9.

8 N. Ahuja, A. K. Chopra and A. A. Ansari, A.A, *IOSR J. Environ. Sci. Toxicol. Food Technol.*, 2016, **10**, 04–14.

9 A. M. Gamal, S. A. Abo-Farha, H. B. Sallam, G. E. A. Mahmoud and L. F. M. Ismail, *Nat. Sci.*, 2010, **8(11)**, 95–110.

10 M. M. Galangash, Z. N. Kolkasaraei, A. Ghavidast and M. Shirzad-Siboni, *Desalin. Water Treat.*, 2018, **116**, 317–328.

11 Y. Huo, H. Wu, Z. Wang, F. Wang, Y. Liu, Y. Feng and Y. Zhao, *Colloids Surf. A Physicochem. Eng. Asp.*, 2018, **549**, 174–183.

12 A. N. M. Salem, M. A. Ahmed and M. F. El-Shahat, *J. Mol. Liq.*, 2016, **219**, 780–788.

13 Xu, H. Zheng, H. Zhou, Y. Wang, K. Luo, C. Zhao, Y. Peng and X. Zheng, *J. Mol. Liq.*, 2018, **256**, 424–432.
